# The Lack of the Essential LptC Protein in the Trans-Envelope Lipopolysaccharide Transport Machine Is Circumvented by Suppressor Mutations in LptF, an Inner Membrane Component of the *Escherichia coli* Transporter

**DOI:** 10.1371/journal.pone.0161354

**Published:** 2016-08-16

**Authors:** Mattia Benedet, Federica A. Falchi, Simone Puccio, Cristiano Di Benedetto, Clelia Peano, Alessandra Polissi, Gianni Dehò

**Affiliations:** 1 Dipartimento di Bioscienze, Università degli Studi di Milano, Milan, Italy; 2 Scuola di Dottorato in Medicina Molecolare e Traslazionale, Università degli Studi di Milano, Segrate, Italy; 3 Istituto di Tecnologie Biomediche, Consiglio Nazionale delle Ricerche, Milan, Italy; 4 Dipartimento di Biotecnologie e Bioscienze, Università degli Studi di Milano-Bicocca, Milan, Italy; Centre National de la Recherche Scientifique, Aix-Marseille Université, FRANCE

## Abstract

The lipopolysaccharide (LPS) transport (Lpt) system is responsible for transferring LPS from the periplasmic surface of the inner membrane (IM) to the outer leaflet of the outer membrane (OM), where it plays a crucial role in OM selective permeability. In *E*. *coli* seven essential proteins are assembled in an Lpt trans-envelope complex, which is conserved in γ-Proteobacteria. LptBFG constitute the IM ABC transporter, LptDE form the OM translocon for final LPS delivery, whereas LptC, an IM-anchored protein with a periplasmic domain, interacts with the IM ABC transporter, the periplasmic protein LptA, and LPS. Although essential, LptC can tolerate several mutations and its role in LPS transport is unclear. To get insights into the functional role of LptC in the Lpt machine we searched for viable mutants lacking LptC by applying a strong double selection for *lptC* deletion mutants. Genome sequencing of viable Δ*lptC* mutants revealed single amino acid substitutions at a unique position in the predicted large periplasmic domain of the IM component LptF (LptF^SupC^). In complementation tests, *lptF*^SupC^ mutants suppress lethality of both Δ*lptC* and *lptC* conditional expression mutants. Our data show that mutations in a specific residue of the predicted LptF periplasmic domain can compensate the lack of the essential protein LptC, implicate such LptF domain in the formation of the periplasmic bridge between the IM and OM complexes, and suggest that LptC may have evolved to improve the performance of an ancestral six-component Lpt machine.

## Introduction

Lipopolysaccharide (LPS), the major glycolipid in the outer layer of Gram-negative bacteria outer membrane (OM), is synthesized at the level of the inner membrane (IM) to be then transported to its final destination (reviewed by [1–3]). In *Escherichia coli*, where this process has been best characterized, the LPS transporter (Lpt) exhibits the overall organization of a trans-envelope ATP-binding cassette (ABC) transporter [4] composed by seven proteins, LptA through LptG, which co-sediment in a membrane fraction that contains both IM and OM and co-purify as a single complex spanning the cytoplasmic, IM, periplasmic and OM cell compartments [5].

LptC, LptA, and LptB are encoded, in this order, as the three promoter-distal genes of the six-cistrons *yrbG* operon, in which *lptC* and *lptA* overlap for 32 nucleotides. In addition to the strong *yrbGp* promoter, two minor promoters (*lptAp1*-*p2*) are located upstream of *lptA* within *lptC* [6,7]. Although *lptAp1* requires σ^E^, this promoter is not activated by several extra-cytoplasmic stress conditions known to induce the σ^E^-dependent promoters, whereas it responds to conditions affecting lipopolysaccharide biogenesis such as depletion of LptC or LptAB, thus implying a specialized σ^E^-dependent LPS stress signaling pathway [7,8]. A bicistronic operon encodes *lptF* and *lptG* [9], whereas *lptD* and *lptE* map at unlinked loci [10–13]. Genetic and biochemical evidence indicate that each of the proteins composing the transenvelope complex is essential for cell viability and that the LPS transporter operates as a single device. In fact, depletion of any Lpt protein, using arabinose dependent conditional expression mutants, leads to similar phenotypes, namely cell lethality, LPS accumulation in the periplasmic leaflet of the IM, and abnormal envelope morphology [9,14,15].

The seven Lpt components form the IM ABC transporter (LptBFGC) and the OM translocon (LptDE), which are connected with each other across the periplasm by LptA. LptF and LptG [9] are IM proteins with six predicted transmembrane segments and a C-terminus located in the cytoplasm [9,16]. Unlike the other components of the Lpt complex, structural information for these two proteins is still lacking. LptF and LptG are thought to form the dimeric IM core of the ABC transporter and have been shown to form a complex with a dimer of the ABC protein LptB, which binds and hydrolyzes ATP, at the cytoplasmic side [9,17,18]. LptB 3D structure exhibits an overall fold resembling the NBD (nucleotide binding domain) proteins, with a RecA-like and an α-helical domain [19,20].

The LptB_2_FG IM sub-complex, which provides energy to LPS transport system through the LptB ATPase activity [21], is connected to the LptDE OM sub-complex across the periplasm through LptC and LptA proteins [14,22,23]. LptC is an IM bitopic protein with a predicted trans-membrane helical domain and a periplasmic region of about 175 amino acids [24], whereas LptA is a periplasmic protein of about 150 residues [25,26]. LptA and the LptC periplasmic domain share very little amino acid sequence conservation (about 13% identity); nevertheless, comparison of their 3D structures reveals a remarkably conserved fold based on a slightly twisted β-jellyroll, composed of 16 (LptA) or 15 (LptC) antiparallel β-strands [24,27,28]. Likewise, although sharing modest sequence identity (~24%), LptA of *E*. *coli* and *Pseudomonas aeruginosa* 3D structures are largely superimposable and the latter can functionally complement *E*. *coli* Δ*lptA* mutants, thus indicating that, despite the scanty sequence homology, the xenogeneic protein properly interacts with the other components in an Lpt hybrid machine [29].

Concentration-dependent LptA oligomerization has been observed in solution [30–32] and, in the crystal, the C-terminal β-strand of one protomer interacts with the N-terminal β-strand of an adjacent molecule [27]. LptA-LptC interactions have also been shown to occur *in vitro* [22] and *in vivo* [23], where the C-terminal β-strand of LptC is predicted to form an interface with the N-terminal β-strand of LptA [23]. Both LptC and LptA have been shown to bind LPS, with LptC that binds with lower affinity than LptA. This is consistent with the idea that LPS transits across the periplasm, passing from the β-jellyroll fold of LptC to that of LptA [21,24,26,33].

Interestingly, the twisted β-jellyroll conformation of LptA and LptC is also conserved by the N-terminal region of LptD [24,27,29,34]. The β-barrel protein LptD and the associated lipoprotein LptE form the OM sub-complex of the LPS transporter [35–37]. The solved crystal structures of the LptDE sub-complex from *Salmonella enterica* sv. Typhimurium and *Shigella flexneri* reveals an unprecedented β-barrel and plug architecture, in which LptD forms a 26-stranded β-barrel that surrounds the LptE plug [34,38]. It has been suggested that the N-terminal domain of LptD provides a hydrophobic intramembrane hole for the transit of the lipid A moiety of the LPS, whereas the hydrophilic polysaccharide moiety is translocated through the luminal gate and a lateral opening of the LptD β-barrel with the assistance of LptE [39]. The structure similarity shared by the LptD N-terminal domain, LptA, and LptC [24,27–29,34] suggests that these proteins, by interacting with each other, may form a hydrophobic groove that accommodates the lipid moiety of LPS for its transport from the inner membrane to the outer membrane [3,21,23,24,39]. It thus appears that the β-jellyroll fold could provide both the hydrophobic environment for the LPS lipid moiety and the interfaces for the interactions of different Lpt components.

LptC specific role and mechanism in LPS transport and the nature of the interaction between the periplasmic and OM components with the IM sub-complex, however, remain unclear. Deletion of its transmembrane N-terminal domain is viable and does not impair LPS transport and LptC assembly with the LptBFG IM complex, although the LptC periplasmic domain lacking the TM domain seems to interact with the IM complex less efficiently than the wild type protein or a chimera with a heterologous TM domain. Point mutations in the N-terminal periplasmic region (G56V) or at the C-terminus (G153R) are detrimental for growth [22,28]. The latter observation apparently contrasts with the fact that deletion of LptC C-terminus is not lethal, although the level of LptB required for viability of the deletion mutant appears to be higher than that required for the wild type *lptC* [40,41].

Considering the dispensability of LptC transmembrane domain and the high structural similarity between its periplasmic domain and LptA, we asked whether some functional redundancy between these structurally analogous components of the Lpt machine could be revealed by testing, under very stringent selective conditions, the essentiality of *lptC* and *lptA*. We obtained eleven independent viable clones lacking *lptC* all of which harbored a suppressor mutation in LptF Arg212, a residue in the predicted periplasmic domain of this integral IM component of the Lpt machine [28]. This finding implies that, with a very specific modification in the predicted periplasmic domain of the IM sub-complex, a six-component Lpt machine may be functional and opens new scenarios for the understanding of the mechanism and evolution of the LPS transport system.

## Materials and Methods

### Bacterial strains and plasmids

**Fig 1:**
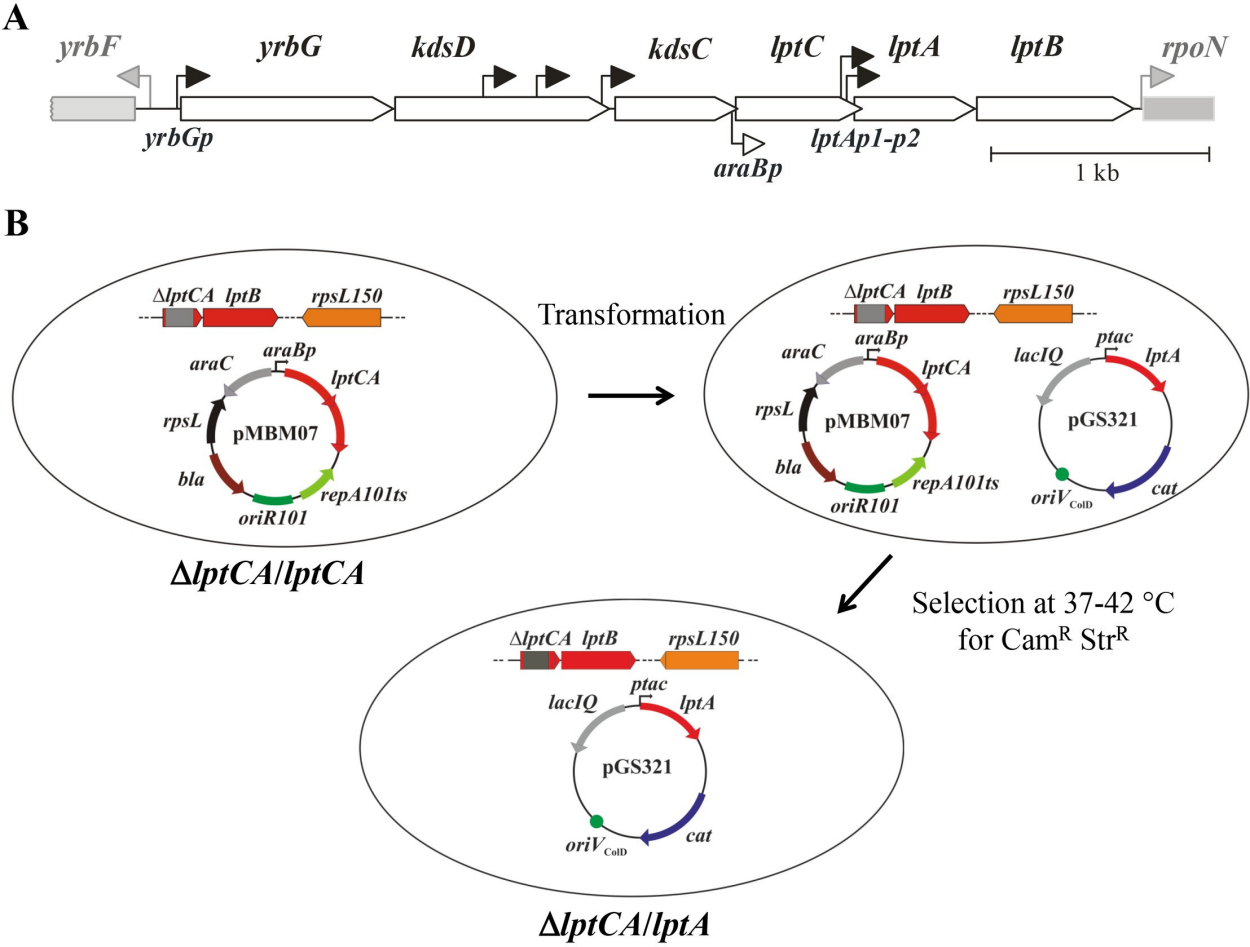
Selection of Δ*lptC* mutants by plasmid shuffling. **A**. Map of the *E*. *coli yrbG-lptB* locus. Coding sequences (open large arrows) are drawn to scale (see 1 kb bar) based on *E*. *coli* MG1655 sequence (GenBank NC_000913). Promoters of the locus [6,7] are indicated by bent arrows with closed arrowheads. Genes and promoters bracketing the *yrbG-lptB* locus are in gray. The insertion point of the *araBp* cassette in the *lptC* depletion mutant FL905 [14] is indicated by the bent arrow with open arrowhead below the map. **B**. Schematic of the plasmid shuffling system by double selection against the resident plasmid. The relevant chromosomal (linear drawings) and plasmid (circles) genotypes are depicted. Δ*lptCA* allele [29] is a short ORF (red arrow) composed by the first (ATG) codon of *lptC*, 27 codons from the FRT sequence (scar [52], gray bar within the ORF) and the 7 terminal codons of *lptA*. The pGS321 (*lptA*) chasing plasmid is depicted as an example. See text for details.

The bacterial strains and plasmids used in this work are listed in S1 and S2 Tables, respectively, with a brief outline of their construction by standard genetic and cloning techniques. Oligonucleotides used in strain and plasmid constructions are listed in S3 Table. All plasmid-cloned DNA regions obtained by PCR were sequenced to rule out the presence of mutations. KG-286.01/pGS104 harbors a chromosomal deletion of *lptC lptA* genes (Δ*lptCA*) and the *lptCAB* genes ectopically expressed from plasmid pGS104 under the *ptac* promoter [29]. In this strain chromosomal *lptB* expression is driven by the main *yrbGp* promoter (see Fig 1). KG-286.01 derivatives harboring plasmids other than pGS104 were obtained by plasmid shuffling (see below and S1 Table).

Unless otherwise stated, bacterial cultures were grown at 37°C in LB [42] or LD [43] medium, supplemented, when required, with 0.2% arabinose, 0.2% glucose, 100 μg/ml ampicillin, 34 μg/ml chloramphenicol, 50 μg/ml kanamycin, 50 μg/ml streptomycin, and 0.1 mM IPTG. Solid media contained 1% agar. Genomic and plasmid DNA was extracted using commercial DNA extraction kits.

### Plasmid shuffling and strain characterization

Plasmid shuffling experiments were performed according to two different approaches. The first one is based on double positive selection against the resident plasmid and selection for the compatible chasing plasmid (Fig 1B and S1 Table) [29]. The bacterial host (KG-286.05/pMBM07, an MC4100 derivative with the chromosomal deletion of two essential genes, *lptC* and *lptA*) harbors on the chromosome the recessive *rpsL150* allele, which confers streptomycin resistance (Str^R^); the resident plasmid pMBM07, an *oriR101* replicon unable to replicate at temperatures ≥ 37°C due to the *repA101*^ts^ mutation, harbors the dominant *rpsL*^+^ allele, a selectable Amp^R^ marker (*bla*), and the *araBp*-*lptCA* cassette for arabinose-dependent complementation of the chromosomal deletion. The parental strain was thus grown at 28°C in LB supplemented with arabinose and ampicillin; electrocompetent cells were transformed by electroporation with the chasing plasmid, a compatible plasmid (*oriV*_ColD_) harboring the selectable Cam^R^ marker *cat* and different combinations of *lptC*, *lptA*, and *lptB* genes. After 1.5 h incubation at 37°C in LB to prevent replication of the resident plasmid and allow expression of the incoming plasmid markers, the culture was plated and incubated at either 37 or 42°C in LB plates supplemented with glucose (to fully repress the *araBp*-*lptCA* cassette expression), chloramphenicol and streptomycin (to select for transformants by the chasing plasmid that had lost pMBM07). Transformants were then screened for Amp^S^ by replica plating on LB glucose plates supplemented with ampicillin and chloramphenicol. The second plasmid shuffling procedure, used for some strain construction (S1 Table), is based on the spontaneous random segregation of the incompatible resident and chasing plasmids, both harbouring the *oriV*_ColD_ replication origin and each expressing a different antibiotic resistance marker (*e*. *g*. Cam^R^ and Kan^R^, respectively) with selection for the chasing plasmid. The parental strain was grown in LB with chloramphenicol at 37°C, electroporated with the chasing plasmid, incubated 1.5 h in LB and plated on LB plates with kanamycin. Loss of Cam^R^ was screened by replica plating. Screening for the presence/absence of *lptC* and *lptA* were performed by PCR with primers FG2760- FG2761 and FG2762- FG2763, respectively. To assess the *lptF* allele harbored by individual clones, sequencing of PCR amplicons obtained with primers AP313- AP316 was performed. Southern blot analysis of genomic DNA and LPS extraction and analysis were performed as previously described [25,44]. The DNA probe for Southern blotting, which covered *lptC* from nucleotide 4 to 532, was obtained by PCR amplification with primers FG3129-FG3130. LPS was fractionated by Tricine-SDS-PAGE (20% polyacrylamide), blotted and decorated with LPS-antibodies as previously described [14].

### Electron microscopy

Bacterial samples were pelleted in Eppendorf tubes, washed with cacodylate buffer 0.2 M (pH 7.4) and fixed with 2% glutaraldehyde in 0.1 M cacodylate buffer. Samples were then post-fixed with 1% osmium tetroxide in 0.1 M cacodylate buffer, dehydrated in a graded ethanol series and embedded in an Epon-Araldite mixture according to standard TEM methods. Ultrathin sections (~50 nm) were cut with a Reichert-Jung ULTRACUT E using diamond knives (DIATOME Ultra 45°). Ultrathin sections, collected on 300 mesh copper grids, were stained with aqueous uranyl acetate and lead citrate carbon coated under a EMITECH K400X carbon coater and observed with a Jeol 100 SX electron microscope. Micrographs were taken directly under the microscope by Kodak 4489 photographic films for TEM.

### Genomic DNA sequencing and data analysis

The library for genomic DNA sequencing was prepared according to the TruSeq DNA Sample preparation protocol (Illumina). Briefly, 1 μg of genomic DNA was sonicated to fragments with a medium length of 400 bp; after end repair, indexed adapters were ligated at DNA fragment ends, libraries were quantified using quantification Real Time PCR (qPCR) by KAPA Library Quant Kits (KAPA Biosystems). After a short amplification step the library was sequenced on an Illumina MiSeq Desktop Sequencer sequencer to generate 300 bp paired-end reads. Raw reads were individually mapped to *E*. *coli* BW2952 genome [45] (NC_012759.1) using the accurate alignment BWA mem algorithm [46] allowing 5% error; removal of duplicated reads was performed with SAMtools; only high quality reads having Q>30 were used for the analysis of variant detection. Single nucleotide variants (SNVs) and short insertions and deletions (indels) detection was performed with SAMtools and Bcftools [47]. A VCF file, containing all the variants for each sample relative to E. *coli* BW2952 was obtained and filtered for low quality variants. SNV having a coverage lower than five high quality reads (Q < 30) and Phred-scaled probability (https://samtools.github.io/hts-specs/VCFv4.2.pdf‬‬‬‬‬‬‬‬‬‬‬‬‬‬‬‬‬‬‬‬‬‬‬‬‬‬‬) lower than 105 (QUAL < 105) were considered low quality variants and discarded. Predicted indels having a coverage lower than six high quality reads (Q < 30) were discarded. Both high quality SNVs and indels were subsequently annotated using SNPeff version 4.0 [48] to determine their effect on coding sequences. Reads mapping statistics are summarized in S4 Table. The assembly of genomic sequences was performed using Velvet 1.2.10 [49] by running the command with 20 different *k*-mers lengths (*k*) using VelvetOptimiser [50] and setting up the following parameters: minimum contig length 500 bp, expected coverage automatic. The assembly metrics were obtained from the Velvet output. Genomic data are available in Sequence Read Archive (SRA, http://www.ncbi.nlm.nih.gov/sra), accession number SRP074314. ‬‬‬‬‬‬‬‬‬‬‬‬‬‬‬‬‬‬‬‬‬‬‬‬‬‬‬‬‬‬‬‬‬‬‬‬‬‬‬‬‬‬‬‬‬‬

## Results

### Isolation of *E*. *coli*Δ*lptC* mutants

In *E*. *coli lptC* is an essential gene, as LptC-depleted cells in arabinose-dependent *lptC* conditional expression mutants are unviable [14]. However, *E*. *coli* tolerates large variations of this protein, as i) mutants lacking the LptC N-terminal transmembrane domain can ectopically complement an *lptC* conditional mutant in the non-permissive condition [28]; ii) *lptC*^Δ139–191^ (a C- terminal deletion mutant), the highly divergent *P*. *aeruginosa lptC* gene, and several *E*. *coli*-*P*. *aeruginosa* chimeric genes can complement LptC-depleted *E*. *coli* cells, albeit under particular conditions of *lptB* expression [41]. In fact, upon LptC depletion in arabinose-dependent conditional expression mutants, *lptAB* expression is driven only by the ancillary promoters *lptAp1* and *lptAp2* [7], as transcription from the main strong promoter *yrbGp* is interrupted by the *araBp* cassette [6] (Fig 1A). In such a condition, complementation by *Pa-lptC* and the chimeric and C-terminally truncated alleles occurs only if a level of *lptB* expression higher than that granted by the ancillary promoters *lptAp1*-*p2* is ectopically provided [41].

To stringently assess whether LptC or any LptC domain is strictly essential for *E*. *coli* viability, we attempted to isolate mutants lacking *lptC* from an ectopically complemented *lptC-lptA* deletion mutant using a double positive selection for the loss of the complementing plasmid coupled to the replacement of the resident plasmid with a chasing plasmid harboring *lptA* only. The plasmid shuffling system, previously described [29], is outlined in Fig 1B and in Materials and Methods. Briefly, the parental strain KG-286.05/pMBM07 harbors on the chromosome the *rpsL150* allele (which confers streptomycin resistance, Str^R^) and the deletion of the overlapping *lptC* and *lptA* genes (Δ*lptCA*) replaced by a short ORF; the downstream *lptB* gene expression is thus driven by the principal *yrbGp* promoter (Fig 1A) whereas the Δ*lptCA* deletion is ectopically complemented by the *lptCA* genes on pMBM07, a thermosensitive-replication plasmid that cannot be maintained at temperatures ≥ 37°C. This plasmid also carries a selectable ampicillin resistance (Amp^R^) marker (*bla*) and the dominant *rpsL*^+^ allele [51], which confers streptomycin sensitivity (Str^S^) to the otherwise Str^R^ host. This strain was transformed with derivatives of the non-thermosensitive, compatible plasmid pGS100, which confers chloramphenicol resistance (Cam^R^), harboring *lptA* (pGS321) or *lptAB* (pGS416), to provide different levels of *lptB* expression (Fig 1B).

Likewise, we also tested whether strains missing *lptA* could be obtained by transforming KG-286.05/pMBM07 with plasmids harboring either *lptC* (pGS402) or the viable *malE-lptC* allele (pGS420), which encodes an LptC derivative lacking the transmembrane domain [28]. A plasmid harboring *lptCA* (pGS404) and the empty vector (pGS401) were used as positive and negative controls, respectively. Loss of the resident plasmid was induced by incubating the transformed cultures at 37°C whereas selection for clones harboring the transforming plasmid (Cam^R^) and missing the resident plasmid (Str^R^) was performed by plating aliquots of the transformed culture at 37 and 42°C on LB glucose agar supplemented with chloramphenicol and streptomycin. The loss of Amp^R^, the selective marker of the resident plasmid pMBM07, was then screened by replica plating.

**Fig 2:**
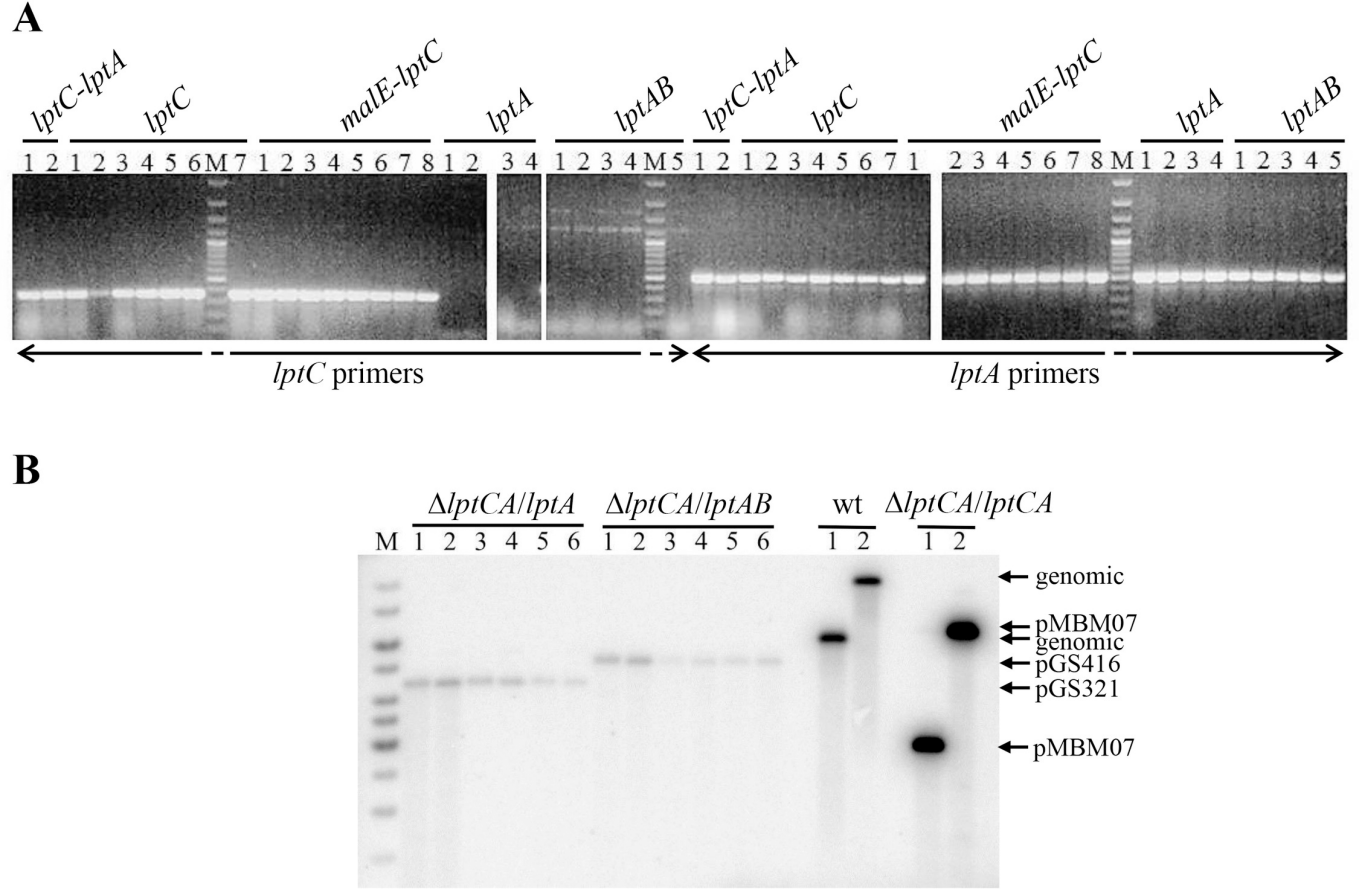
Screening for the presence of *lptA* and *lptC* in plasmid shuffled clones by PCR and Southern blotting analysis. **A**. Electrophoretic analysis of PCR amplicons obtained with *lptC*- and *lptA*-specific pairs of primers (FG2760- FG2761 and FG2762- FG2763, respectively), as indicated on the bottom of the panel, from plasmid shuffled and control strains. On top of the lanes the *lpt* genes harbored by the plasmid used for transformation of the parental KG-286.05/pMBM07 are indicated: *lptC-lptA*, lanes 1–2, pGS404; *lptC*, lanes 1–7, pGS402; *malE-lptC*, lanes 1–8, pGS420; *lptA*, lanes 1–4, pGS321; *lptAB*, lanes 1–5, pGS416; M, molecular weight markers (100 bp ladder). See text for details. **B**. Southern blotting of DNA from plasmid shuffled transformant clones. Total DNA from clones obtained by shuffling with the plasmid indicated on top of the panel was digested with *Sal*I (odd lane numbers) or *Hind*III (even lane numbers), Southern blotted and hybridized with a radioactive DNA probe obtained with primers FG3129-FG3130 and covering *lptC* nucleotides 4–532. Δ*lptCA*/*lptA*, lanes 1–6, six purified KG-286/pGS321 clones; Δ*lptCA*/*lptAB*, lanes 1–6, six purified KG-286/pGS416 clones; wt, lanes 1–2, AM604; Δ*lptCA*/*lptCA*, lanes 1–2, parental strain KG-286.05/pMBM07; M, molecular weight markers (1 kb ladder). The bands in lanes M, Δ*lptCA*/*lptA* 1–6, and Δ*lptCA*/*lptAB* 1–6 are due to non-specific hybridization of the probe with the DNA marker (M) and with the chasing plasmids (pGS321 and pGS416), both of which were linearized by *Sal*I and *Hind*III digestions.

Cam^R^ Str^R^ Ts^+^ transformants were obtained, as detailed in Materials and Methods, with both pGS321 (*lptA*) and pGS416 (*lptAB*) plasmids at frequencies between 0.2 and 0.7 per ng of plasmid DNA (Table 1). Transformation frequency with pGS404 (*lptCA*) was > 3,000 fold higher, whereas no transformants (< 0.25/ng plasmid DNA) were obtained with the empty vector pGS401. Four out of four and five out of five clones tested among those obtained by transformation with pGS321 and pGS416, respectively, were found to be Amp^S^. To rule out the presence of a displaced *lptC* gene in such mutants putatively lacking *lptC*, we performed on a number of purified plasmid-shuffled clones both PCR analysis with an *lptC*-specific pair of primers and Southern blot analysis with an *lptC*-specific probe covering nucleotides 4–532 of *lptC*. No signal of *lptC* presence could be detected by either approach in all clones tested (Fig 2), which thus represent *bona fide* viable *E*. *coli* mutants lacking LptC. It should be noted that all clones analyzed, irrespective of the chasing plasmid used or the selection temperature, derive from samples of the same parental KG-286.05/pMBM07 culture and thus cannot be considered *a priori* independent mutant clones.

**Table 1:** Frequency of transformants^a^ upon selection for the chasing plasmid at non-permissive temperature for the resident plasmid complementing the Δ*lptCA* mutation^b^.

Chasing Plasmid	*lpt* genes on plasmid	Selection	
		42°C Str^R^ Cam^R^	37°C Str^R^ Cam^R^
pGS401	none	< 0.25	< 0.25
pGS404	*lptC-lptA*	> 2,000	> 2,000
pGS321	*lptA*	0.2	0.5
pGS416	*lptAB*	0.7	0.7
pGS402	*lptC*	0.7	1.1
pGS420	*malE-lptC*	1.8	0.9

^a^ n. of transformants per ng of plasmid DNA in the described selective conditions

^b^ recipient strain KG-286.05/pMBM07

Cam^R^ Str^R^ Ts^+^ transformants were also obtained at 37 and 42°C at frequencies between 0.7 and 1.8 transformants/ng of plasmids DNA with pGS402 (*lptC*) and pGS420 (*malE-lptC*). However, PCR analysis with *lptC*-specific primers revealed the presence of *lptA* in all fifteen clones tested (Fig 2A), including six Amp^S^ clones, thus suggesting that variable portions of pMBM07 containing *lptA* were maintained, either as plasmids or integrated, in the genome of the selected transformants. Thus viable Δ*lptA* mutants could not be obtained under our experimental conditions.

### Phenotypic characterization of *E*. *coli*Δ*lptC* mutants

**Fig 3:**
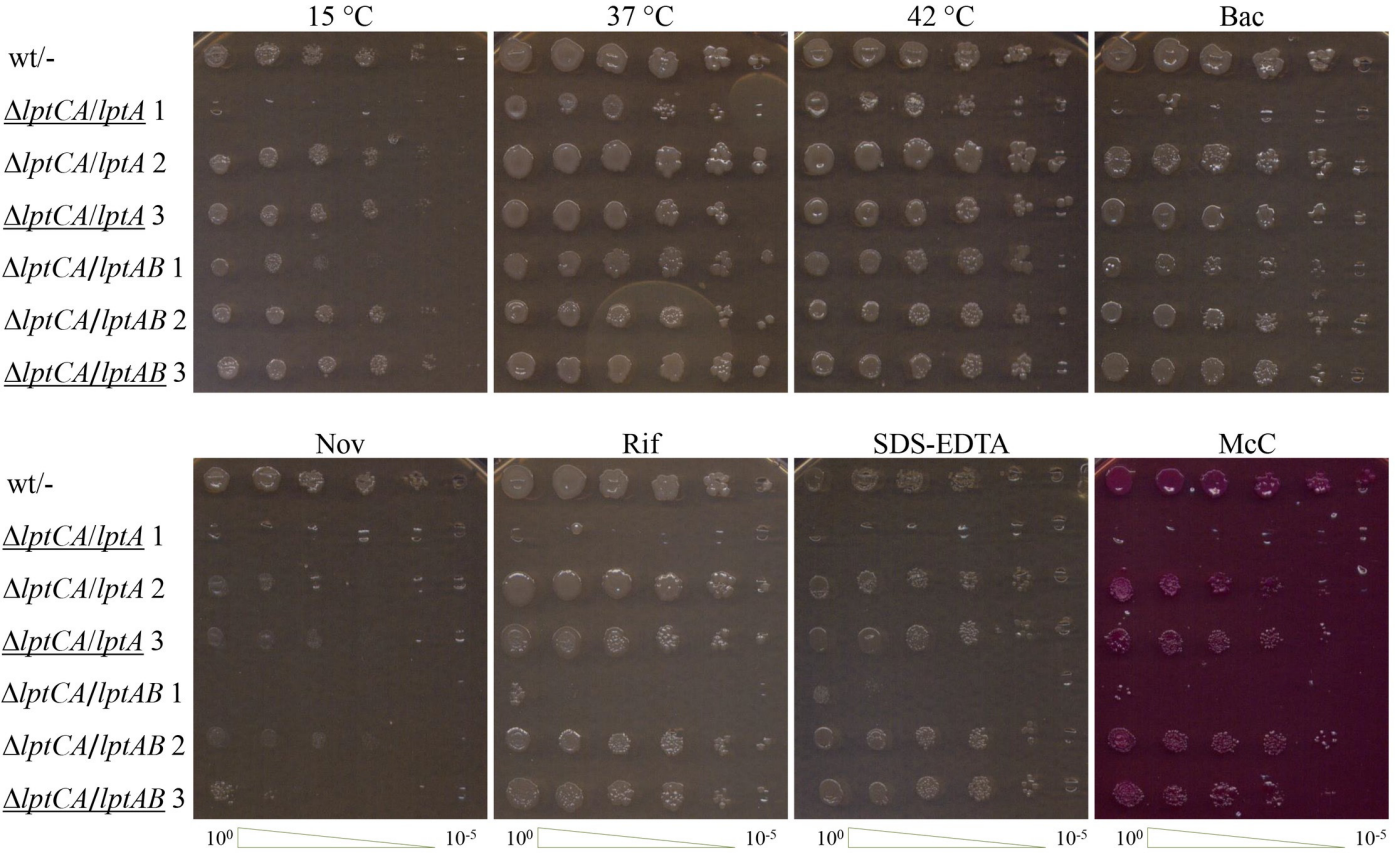
Phenotypic analysis of Δ*lptC* mutants. Bacterial cultures grown in LB-glucose-chloramphenicol at 37°C were serially diluted 1:10 in microtiter wells and replica plated in LB agar plates all supplemented with glucose and chloramphenicol and, where indicated, bacitracin (50 μg/ml), novobiocin (10 μg/ml), rifampicin (2.5 μg/ml) or SDS-EDTA (0.5% and 0.25mM, respectively). MacConkey agar plates were supplemented with glucose and chloramphenicol. The plates were incubated overnight at 37°C (or 42°C, where indicated) or 3 d at 15°C, as indicated on top of the pictures. The serial dilutions are indicated on the bottom. Strains are indicated on the left of the panels by the relevant chromosomal mutant allele(s) followed by a slash and the plasmid encoded allele(s); the clones selected for whole-genome sequencing (see below) are underlined and the mutation found in *lptF* is reported in the legend. wt/-, AM604/pGS401; Δ*lptCA*/*lptA* 1, KG-292.01/pGS321 (*lptF*^R212C^); Δ*lptCA*/*lptA* 2, KG-286.10/pGS321; Δ*lptCA*/*lptA* 3, KG-293.01/pGS321 (*lptF*^R212S^); Δ*lptCA*/*lptAB* 1, KG-286.13/pGS416; Δ*lptCA*/*lptAB* 2, KG-286.14/pGS416; Δ*lptCA*/*lptAB* 3, KG-294.01/pGS416 (*lptF*^R212S^).

**Fig 4:**
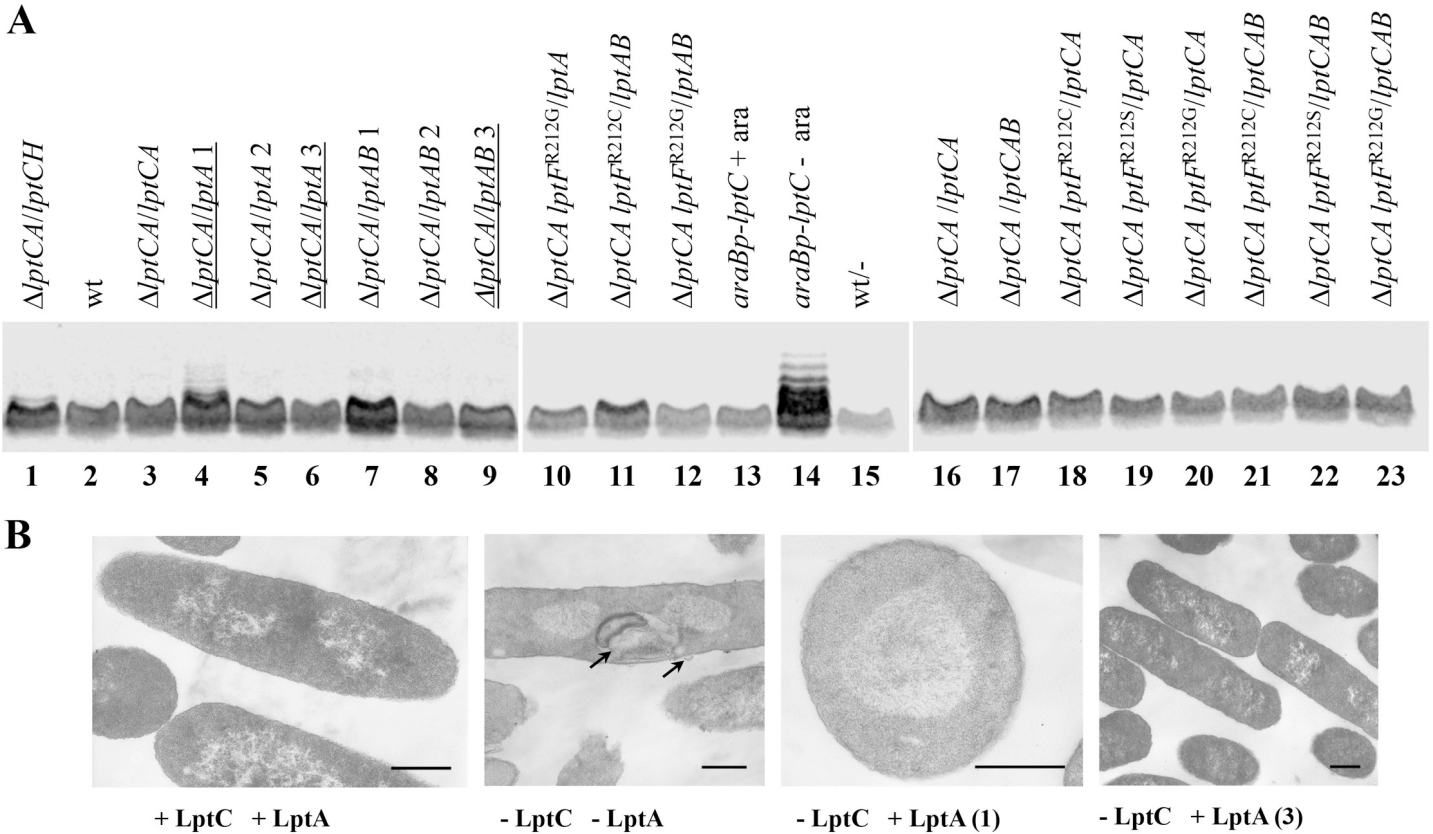
LPS analysis in Δ*lptC* mutants and electron microscopy. **A**. Unless otherwise indicated, bacterial cultures were grown at 37°C in LB glucose plus the antibiotic required to select for plasmid maintenance up to an OD_600_ of 0.6 and LPS was extracted, fractionated by gel electrophoresis and immunoblotted as described in Materials and methods. All samples were loaded onto three gels (corresponding to the three panels of the figure) and run in parallel for 5.5 h at 18 mA/gel. Lanes were loaded with LPS extract aliquots corresponding to an OD_600_ of 0.2. Strains are indicated on top of the panels as detailed in Fig 3. Left panel: 1, Δ*lptCA*/*lptCH*, KG-286.07/pGS406; 2, wt, AM604; 3, Δ*lptCA*/*lptCA*, KG-286.05/pMBM07, grown at 28°C with arabinose; 4, Δ*lptCA*/*lptA* 1, KG-292.01/pGS321 (*lptF*^R212C^); 5, Δ*lptCA*/*lptA* 2, KG-286.10/pGS321; 6, Δ*lptCA*/*lptA* 3, KG-293.01/pGS321 (*lptF*^R212S^); 7, Δ*lptCA*/*lptAB* 1, KG-286.13/pGS416; 8, Δ*lptCA*/*lptAB* 2, KG-286.14/pGS416; 9, Δ*lptCA*/*lptAB* 3, KG-294.01/pGS416 (*lptF*^R212S^). Central panel: 10, Δ*lptCA lptF*^R212G^/*lptA*, KG-295.01/pGS321; 11, Δ*lptCA lptF*^R212C^/*lptAB*, KG-297.01/pGS416; 12, Δ*lptCA lptF*^R212G^/*lptAB*, KG-296.01/pGS416; 13, *araBp-lptC* + ara, FL905 grown with arabinose; 14, *araBp-lptC*—ara, FL905 upon LptC depletion without arabinose; 15, wt/-, AM604/pGS401. Right panel: 16, Δ*lptCA*/*lptCA*, KG-286.06/pGS404; 17, Δ*lptCA*/*lptCAB*, KG-286.01/pGS104; 18, Δ*lptCA lptF*^R212C^/*lptCA*, KG-292.02/pGS308; 19, Δ*lptCA lptF*^R212S^/*lptCA*, KG-293.02/pGS308; 20, Δ*lptCA lptF*^R212G^/*lptCA*, KG-295.02/pGS308; 21, Δ*lptCA lptF*^R212C^/*lptCAB*, KG-297.02/pGS305; 22, Δ*lptCA lptF*^R212S^/*lptCAB*, KG-294.02/pGS305; 23, Δ*lptCA lptF*^R212G^/*lptCAB*, KG-296.02/pGS305. **B**. Electron micrographs of Δ*lptC* mutants. Bacterial cultures were grown at 37°C in LB glucose plus the antibiotic required to select for plasmid maintenance up to an OD_600_ of 0.5, excepting for the LptC-LptA-depleted parental control KG-286.05/pMBM07, which was grown at 28°C in LB arabinose, spun, resuspended in LB without arabinose, and further incubated 10 h for depletion. 10 OD units were spun and processed for electron microscopy as described in Materials and methods. The nominal magnification is reported in parentheses after the strain name. +LptC +LptA, AM604/pGS401 (20,000 x; wild type control); -LptC -LptA, KG-286.05/pMBM07 (15,000 x; LptC-LptA-depleted control); -LptC +LptA (1), KG292.01/pGS321 (30,000 x, cross section; Δ*lptC* *lptF*^R212C^); -LptC +LptA (3), KG293.01/pGS321 (10,000 x; Δ*lptC* *lptF*^R212S^). The size bar corresponds to 0.5 μm. In image -LptC -LptA a multilayered membranous body and a budding vesicle are indicated by arrows.

Impaired LPS transport may lead to growth defects (such as lower growth rate, cold- and/or thermo-sensitivity), LPS modifications (some of which can be detected by LPS gel electrophoresis), increased sensitivity to toxic chemicals, and/or structural abnormalities of the cell envelope, such as multilayered membranous bodies within the periplasmic space and vesicles budding from the OM [6,14,25,36]. As shown in Fig 3, six different Δ*lptC* clones tested (three complemented by *lptA* and three by *lptAB*) exhibited variable degrees of sensitivity to a set of toxic compounds and two of them did not grow at 15°C, thus suggesting that, although derived from a single parental culture, individual clones might bear different compensatory mutations. Moreover, the cold sensitive clone complemented by *lptA* exhibited an altered LPS electrophoretic mobility pattern resembling the one observed in the *lptC*-depleted control, although the intensity of the LPS ladder bands was less intense in the former (Fig 4A, compare lanes 4 and 14), thus suggesting the presence of modified LPS. In the cold sensitive clone complemented by *lptAB* (lane 7), however, a low mobility signal was barely detectable. On the contrary, no gross structural alterations of the cell envelope could be detected in the two Δ*lptC* mutants examined (Fig 4B, 3rd and 4th micrographs from right), even in the strain that exhibited altered LPS electrophoretic mobility (Fig 4B, 3rd micrograph, cross section), whereas membranous bodies and vesicles resembling those previously described in cells depleted of Lpt components [6,14,25,36] were readily detected in in the LptC-LptA-depleted parental strain (Fig 4B, 2nd micrograph). Generation times of LB-glucose cultures at 37°C of three clones complemented by *lptA* (pGS321) and three by *lptAB* (pGS416) scattered, without any apparent correlation, between 28 min (like the parental AM604/pGS401) and 33 min.

### *E*. *coli* Δ*lptC* mutation is suppressed by amino acid substitutions at a unique residue of LptF

The phenotypic variability exhibited by the different Δ*lptC* isolates suggests that different compensatory suppressor mutations could have been selected during the plasmid shuffling procedure; alternatively, different adaptive regulatory systems could have been activated to overcome the lack of LptC. To identify potential Δ*lptC* suppressor mutations, we sequenced the genome of the parental (KG-286.05/pMBM07) and three Δ*lptC* mutants that exhibited different phenotypes and had been obtained upon shuffling with the plasmid harboring *lptA* or *lptAB* (clones Δ*lptCA*/*lptA* 1, Δ*lptCA*/*lptA* 3, and Δ*lptCA*/*lptAB* 3 in Figs 3 and 4, renamed KG-292.01/pGS321, KG-293.01/pGS321, and KG-294.01/pGS416, respectively). The reads were mapped to the reference strain E.*coli* BW2952 complete genome sequence (Accession number NC_012759.1) [45] giving > 99% coverage (see mapping statistics in S4 Table). Sequence variations between these four strains and the reference BW2952 that mapped in open reading frames are reported in Table 2. Comparison between the Δ*lptC* mutants and their parental KG-286.05/pMBM07 genomic sequences revealed the presence, in each of the three mutants, of a single nucleotide substitution at base 634 of *lptF* (either C➔A transversion or C➔T transition) that caused a single amino acid substitutions at arginine 212 of the encoded LptF protein (R212C in strains KG-292.01/pGS321, and R212S in KG-293.01/pGS321 and KG-294.01/pGS416; Table 2). Strain KG-293.01/pGS321 harbored an additional missense mutation in a small ORF of unknown function (BWG_3693) and a synonymous mutation in the minor tail protein M (BWG_3735) of λ phage, both within the λp*lac*Mu inserted in the parental strain chromosome [45]. All remaining variations within protein coding sequences from the reference genome (4 mutations) were shared by both the parental and the three mutants. Differences in intergenic regions among the four sequenced strains, listed in S5 Table, were clustered in regions harboring rRNA and/or tRNA genes, or within pseudogenes. Additional 35 variations (not listed) from the reference sequence in intergenic regions were shared by both the parental and the mutant strains.

**Table 2:** Mutations in ORFs of parental and Δ*lptC* viable mutants as compared with *E*. *coli* BW2952 sequence.

STRAIN^a^	ORF	Gene	N^b^	Mutation	Position in		Change in		Description
				type	CDS^c^	Codon	Codon	Amino acid	
A, B, C, D	BWG_0606	*aroG-1*	G➔A	missense	655	219	Gcg➔Acg	A➔T	Phosphoglyceromutase 1
A, B, C, D	BWG_1070	*orf*	C	frameshift	156–157	52–53	-	-	Predicted divalent heavy-metal cations transporter
A, B, C, D	BWG_1086	*yciE*	T➔A	missense	388	130	Atc➔Ttc	I➔F	Conserved protein
A, B, C, D	BWG_3107	*insD*	C	frameshift	346–347	116	-	-	IS2 transposase
C	BWG_3693	*orf*	C➔T	missense	103	35	Gac➔Aac	D➔N	Protein of unknown function
C	BWG_3735	λ M	T➔C	synonymous	153	51	ccT➔ccC	P	Polypeptide: Minor tail protein M
B	BWG_3967	*lptF*	C➔T	missense	634	212	Cgc➔Tgc	R➔C	LptF
C, D	BWG_3967	*lptF*	C➔A	missense	634	212	Cgc➔Agc	R➔S	LptF

^a^ A, KG-286.05/pMBM07 (parental); B, KG-292.01/pGS321; C, KG-293.01/pGS321; D, KG-294.01/pGS416

^b^ Base substitution relative to the NC_012759.1 sequence of BW2952.

^c^ CDS, coding sequence

Although harboring a different chasing plasmid, the two *lptF*^R212S^ mutants derive from the same parental culture and thus cannot be considered to bear independent mutations. Anyway, one of the sequenced mutants harbored a different mutation (R212C) in the same residue of LptF, thus being an independent mutant from the same culture. These data strongly suggest that the change of a specific residue in LptF suppresses the lethal phenotype associated with the lack of the essential protein LptC.

To better support this hypothesis and identify other potential Δ*lptC* suppressor alleles of *lptF*, we selected for new independent Δ*lptC* mutants from single-colony cultures of KG-286.05/pMBM07 by plasmid shuffling with pGS321 or pGS416, as described above, obtaining Cam^R^ Str^R^ Ts^+^ transformants at 42°C in nine out of ten cultures tested, four of which harboring *lptA* and five *lptAB* on the chasing plasmid. All nine independent isolates, upon screening for the Amp^R^ marker by replica plating and *lptC* by PCR of the resident pMBM07 plasmid, turned out to be ampicillin sensitive and *lptC*-negative. Sequencing of the *lptF* gene showed that all nine independent transformants harbored a single amino acid substitution at residue R212 (Table 3). In addition to the mutations found in the first round of selection (two new independent R212C and three new independent R212S mutants), three independent R212G amino acid substitution mutants were also obtained. Combining data of the first and second round of selection, however, no specific correlation between a given type of mutant and the presence of either *lptA* or *lptAB* on the chasing plasmid was observed, as any of the three mutations was obtained upon shuffling with either chasing plasmid (Table 3). Two *lptF*^R212G^ mutants tested, one complemented by *lptA* and the other by *lptAB*, and an *lptF*^R212C^ mutant complemented by *lptAB*, did not produced anomalous LPS bands of low electrophoretic mobility (Fig 4A, lanes 10–12).

**Table 3:** Independent^a^ *lptF* mutants that suppress Δ*lptC*.

Suppressor strain	Genes on plasmid^b^	Transform. efficiency^c^	LptF aa change
KG-292.01/pGS321	*lptA*	0.5	R212C
KG-297.01/pGS416	*lptAB*	0.3	R212C
KG-303.01/pGS416	*lptAB*	0.7	R212C
KG-295.01/pGS321	*lptA*	1.5	R212G
KG-299.01/pGS321	*lptA*	0.03	R212G
KG-300.01/pGS321	*lptA*	0.3	R212G
KG-296.01/pGS416	*lptAB*	0.1	R212G
KG-293.01/pGS321*	*lptA*	0.5	R212S
KG-301.01/pGS321	*lptA*	0.3	R212S
KG-294.01/pGS416*	*lptAB*	0.7	R212S
KG-298.01/pGS416	*lptAB*	0.3	R212S
KG-302.01/pGS416	*lptAB*	0.7	R212S

^a^ The two whole-genome sequenced non-independent mutants are marked by an asterisk (*)

^b^ *lptA*, pGS321; *lptAB*, pGS416

^c^ transformants per ng of transforming DNA

### Characterization of LptF mutants suppressing Δ*lptC*

**Fig 5:**
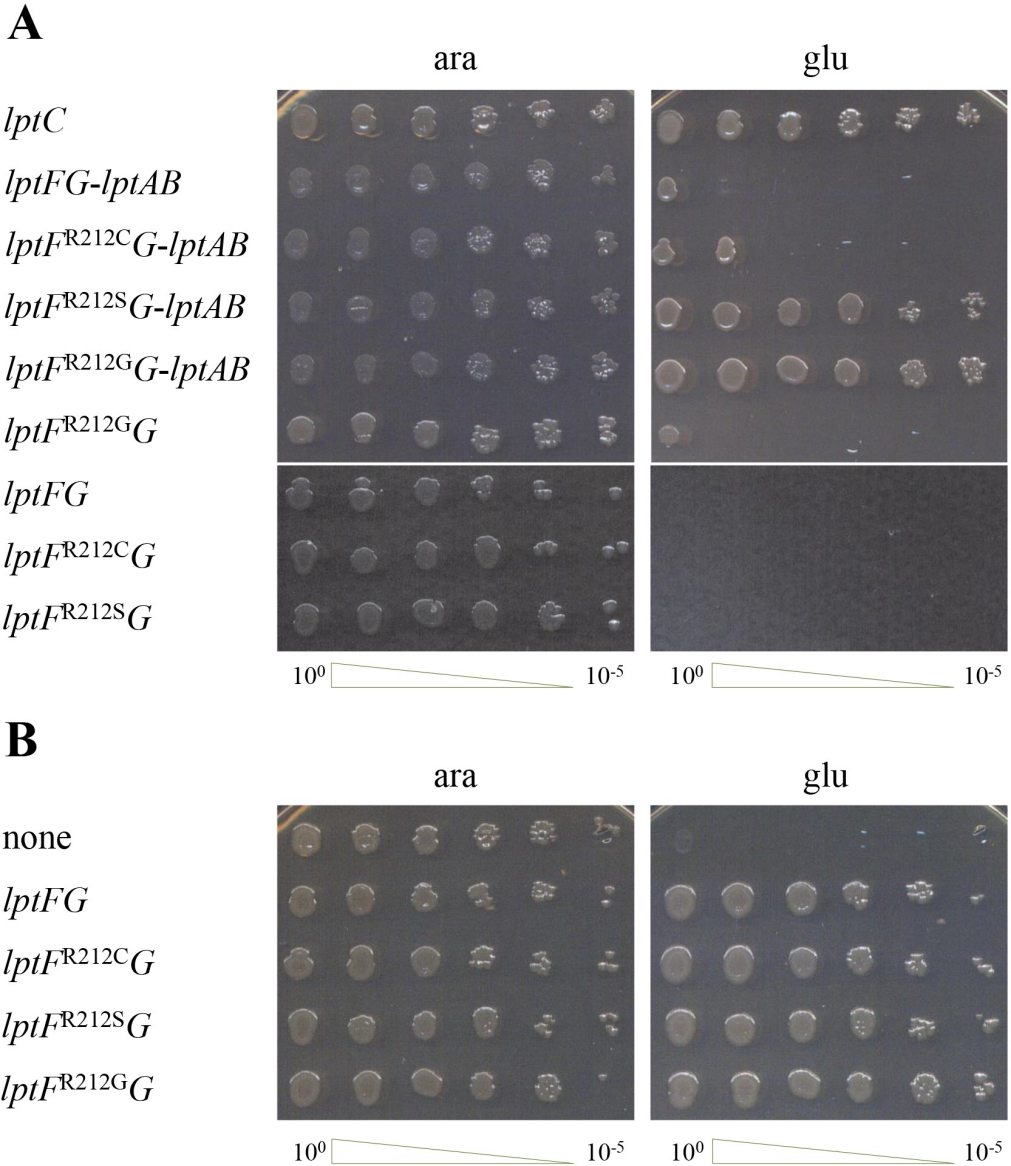
Suppression of LptC depletion and LptC compatibility by *lptF*^SupC^ alleles. Cultures of FL905 (*araBp*-*lptC*) (Panel **A**) and NR1113 (*araBp*-*lptFG*) (Panel **B**) strains transformed with pGS401 derivatives expressing the *lpt* genes listed on the left were grown in LB-arabinose-chloramphenicol, serially diluted 1:10 in microtiter wells, and replica plated in agar plates with either arabinose (ara) to induce or glucose (glu) to fully repress the *araBp* promoter. The serial dilutions are indicated on bottom of the panels.

The presence of specific LptF R212 residue substitutions in all the eleven independent Δ*lptC* mutants isolated strongly indicates that such *lptF* mutations (henceforth collectively designated as *lptF*^SupC^) suppress the lethal phenotype associated to the lack of LptC. However, we could not rule out that undetected additional mutations could contribute to the Δ*lptC* suppressor phenotype selected by plasmid shuffling. In fact, the three whole-genome sequenced mutants harbor mutations in non-coding regions that could not be tested for their contribution to the suppression phenotype; moreover, a portion, albeit small (about 1%), of the genome was not covered by sequencing (S4 and S5 Tables), whereas in the nine additional independent suppressor mutants only *lptF* was sequenced. Therefore, to address whether the different mutations in LptF R212 residue could be sufficient to support the growth of *E*. *coli* in the absence of LptC avoiding selection of additional mutations, we tested whether *lptF*^SupC^ could support the growth of the *lptC* conditional expression mutant FL905 upon LptC depletion. Since, as mentioned above, in the absence of the arabinose inducer the downstream *lptAB* operon can only be transcribed from the ancillary promoters *lptAp1*-*p2*, which might not grant an *lptAB* expression level sufficient for cell viability under critical conditions [7,41], we ectopically expressed in FL905 the *lptF*^SupC^-*lptG* operon either alone or together with *lptAB*. As shown in Fig 5A, the growth defect caused by depletion of LptC (no arabinose) i) in the positive control strain, as previously shown [6], was complemented by ectopically expressed *lptC* both with and without *lptAB* co-expression; ii) was suppressed by *lptF*^R212G^ and *lptF*^R212S^ when coexpressed with *lptAB* but not when expressed alone; and iii) was suppressed by *lptF*^R212C^ in neither condition. As in FL905 a wild copy of *lptF* gene is harbored on the chromosome, the lack of suppression by *lptF*^R212C^ could be a consequence of the recessive nature of the mutant allele, although in higher copy number than the wild type copy. Alternatively, suppression of Δ*lptC* by *lptF*^R212C^ may require additional mutations that are present in the original mutants selected upon plasmid shuffling but not in the *lptC* depletion strain. On the contrary, viability of LptC depleted cells expressing *lptF*^R212G^ or *lptF*^R212S^ clearly indicates that such alleles i) are sufficient to support the growth of a population of LptC-depleted cells, thus without selection of any additional mutation; ii) are dominant (at least when harbored by a plasmid) over the wild type allele; and iii) require expression of *lptAB* at a level higher than that provided by the ancillary promoters *lptAp1*-*lptAp2*.

We also addressed whether *lptF*^SupC^ mutations in the haploid state are compatible with the presence of *lptC*. To this end we replaced by plasmid shuffling in each type of *lptF*^SupC^ mutants the resident plasmid harboring Cam^R^ and either *lptA* or *lptAB* with an incompatible plasmid harboring a different antibiotic resistance marker (Kan^R^) and either *lptCA* or *lptCAB*. Selection of Kan^R^ transformants was done in the absence of chloramphenicol so as to allow segregation of the resident plasmid. Six Kan^R^ transformants for each strain were then colony purified and tested for the presence of the resident plasmid (Cam^R^). As shown in Table 4, none of the strains transformed, as a control, by the chasing plasmid vector without *lpt* genes lost the resident plasmid (0/6 Cam^S^) as it carried genes essential for viability; likewise, the *lptF*^+^ strains transformed by the chasing plasmid with *lptA* or *lptAB* but missing *lptC* did not lose the resident plasmid. On the contrary, all the *lptF*^SupC^ clones segregated a plasmid-shuffled (Cam^S^) progeny. Finally, both *lptF*^+^ and *lptF*^SupC^ strains could be transformed, albeit at different efficiencies, by the chasing plasmid carrying *lptCA* or *lptCAB* and segregated a Cam^S^ progeny. We then assessed by direct sequencing that each type of *lptF*^SupC^ shuffled clones had retained the original *lptF* allele and no reversions or additional mutations had occurred. Therefore, all the three haploid *lptF*^SupC^ mutations are compatible with the presence of LptC. To rule out that compatibility between *lptF*^SupC^ and *lptC* could depend on additional mutations originated upon selection of the *lptF*^SupC^ strains and/or plasmid shuffling to reintroduce *lptC* in the suppressor strains, we transformed with plasmids harboring the *lptFG* operon with the mutant *lptF*^SupC^ alleles the NR1113 strain [9], a conditional mutant which expresses the wild type *lptFG* operon under the arabinose inducible promoter *araBp*. As shown in Fig 5B, each of the three *lptF*^SupC^ mutations was able to complement the wild type LptF-depleted cells. Moreover, in the presence of *lptC* none of the suppressor mutants produced anomalous LPS (Fig 4A, right panel). Overall these data indicate that the *lptF*^SupC^ alleles, which suppress the lack of LptC, are compatible with the presence of LptC.

**Table 4:** *lptF*^SupC^ mutants are compatible with *lptC*.

N	Strain	*lptF*^a^	Resident	*lpt* genes on the chasing plasmid^c^					
			plasmid^b^	none		*lptA(B)*^d^		*lptCA(B)*^d^	
				e.o.t.^e^	Cam^S^	e.o.t.^e^	Cam^S^	e.o.t.^e^	Cam^S^
1	KG-286.06/pGS404	wt	*lptCA*	7.03E+03	0/6	1.77E+04	0/6	1.09E+02	6/6
2	KG-292.01/pGS321	R212C	*lptA*	5.73E+03	0/6	9.37E+03	6/6	8.00E+02	6/6
3	KG-293.01/pGS321	R212S	*lptA*	7.67E+03	0/6	2.87E+04	6/6	6.33E+02	6/6
4	KG-295.01/pGS321	R212G	*lptA*	2.42E+04	0/6	2.47E+04	6/6	1.50E+03	6/6
5	KG-286.04/pGS104	wt	*lptCAB*	5.17E+04	0/6	5.92E+04	0/6	6.05E+02	6/6
6	KG-297.01/pGS416	R212C	*lptAB*	5.87E+03	0/6	6.33E+03	6/6	1.00E+03	6/6
7	KG-294.01/pGS416	R212S	*lptAB*	1.01E+04	0/6	1.74E+04	6/6	2.00E+02	6/6
8	KG-296.01/pGS416	R212G	*lptAB*	1.54E+04	0/6	3.02E+04	6/6	4.67E+02	6/6

^a^ *lptF* allele of the host strain

^b^ *lpt* genes of the resident plasmid

^c^ *lpt* genes of the chasing plasmid; none, pGS303; *lptA*, pGS323; *lptAB*, pGS324; *lptCA*, pGS308; *lptCAB*, pGS305

^d^ pGS323 and pGS308 were used for transformations 1–4, pGS324 and pGS305 for transformations 5–8

^e^ Efficiency of transformation (number of transformants per μg of transforming DNA)

Finally we addressed whether the different suppressor mutations, in association with *lptA* or *lptAB*, both in the absence and in the presence of *lptC*, correlated with a specific phenotype such as cold or toxic compound sensitivity. The results obtained with a set of strains constructed by incompatible-plasmid shuffling are shown in S6 Table. From these data it appears that the *lptF*^R21C2^ allele is the weakest suppressor as it confers i) increased sensitivity to the three antibiotics tested, SDS, and MacConkey, in association with both *lptA* and *lptAB*, and ii) cold sensitivity, only in part alleviated by *lptAB*. The other two suppressor mutants exhibited increased sensitivity to a more limited spectrum of conditions (novobiocin, SDS and MacConkey), in part alleviated by coexpression of *lptAB*. All such defective phenotypes were abolished by expression of *lptC*.

## Discussion

In this work we implemented a double positive selection procedure for gene-deletion mutants to stringently test the essentiality of *lptA* and *lptC*, two genes coding for components of the LPS transport machine that connect the IM and the OM sub-complexes. The selection system is based on a streptomycin resistant (*rpsL*) strain harboring a chromosomal deletion of the target gene ectopically complemented by a thermosensitive plasmid carrying both a copy of the target gene and the dominant wild type *rpsL* allele. High temperature rapidly stops replication of the resident plasmid and thus generates a large population of cells with the non-complemented deletion of the target gene (plasmid-less cells may keep dividing until depletion of the target protein occurs) whereas streptomycin kills the parental cells still carrying the resident complementing plasmid with the dominant *rpsL*^+^ allele. Thus under such conditions viable deletion mutants of a *bona fide* essential gene either cannot be obtained or must harbor additional suppressor mutation(s). In its simple form (*i*.*e*. deletion of a chromosomal gene complemented by a thermosensitive plasmid conferring antibiotic sensitivity) this may represent a straightforward and powerful procedure to systematically assess the essentiality of any gene in suitable bacterial systems under very stringent conditions, and may supersede other "negative" methods based on random or targeted gene disruption (see [40,53]).

We coupled selection for the target gene deletion mutant with plasmid shuffling as we started from a strain harboring a deletion of two essential genes, *lptA* and *lptC*, and thus a chasing plasmid carrying either gene was required for complementation of the non-target gene. In practice, this experimental setup was for two reasons: i) we wanted to stringently test the essentiality of both genes, whose encoded proteins conserve a high structural similarity, in comparable experimental conditions; ii) the two genes constitute a genetically complex locus as they are partially overlapped and ancillary promoters are located within the upstream *lptC* gene (Fig 1A; [7]). Thus transferring the genes on a complementing plasmid could facilitate genetic manipulations of the system. This plasmid shuffling system was originally set up to obtain allelic replacement of *E*. *coli lptA* gene with the homologue from *P*. *aeruginosa* [29]. We argued that, unlike plasmid shuffling based on incompatible plasmids (*i*.*e*. plasmids with the same origin of replication), which rely on random segregation of the resident and chasing plasmids, this system could be suited to assess whether either putative essential gene is essential or not. Our data indicate that this system is sufficiently robust to select for rare suppressor mutants that bypass the loss of an essential gene such as *lptC*, thus providing a valuable tool to identify interactions between essential proteins and cell pathways. It should be noted that after the loss of the resident complementing plasmid the cells may undergo several divisions before depletion of the LptC protein occurs [6] and thus suppressor mutations may have arisen not only during growth of the competent cell culture, but also after transformation with the chasing plasmid at any time before the level of LptC becomes insufficient for cell viability (both during the 1.5 h incubation in liquid and upon plating in solid selective media). Therefore, selection for Δ*lptC* survivors is applied to a bacterial population much larger (although its size cannot be easily assessed) than that initially transformed by the chasing plasmid DNA, thus increasing the chance to find rare mutants.

Genetic and biochemical evidence indicate that LptC is an essential component of the LPS transport machinery and is required for *E*. *coli* viability [6,22,27]. LptC has been thought to connect the IM ABC transporter LptBFG with the periplasmic LptA that, in turn, would interact with the periplasmic N-terminal domain of LptD. LptA, the periplasmic C-terminal domain of the bitopic LptC protein, and the periplasmic LptD N-terminal domain exhibit high structural similarity, the β-jellyroll fold, despite the scarce sequence conservation [24,27–29,34]. It has been suggested that these three elements, by interacting with each other, form a hydrophobic groove that accommodates the lipid moiety of LPS for its transport from the inner membrane to the outer membrane [21,23,24,39]. In this model, however, the connection between the LptC-A-DE complex and the IM LptBFG transporter, which provides energy to the Lpt system by ATP hydrolysis, has not been clarified. The transmembrane domain of LptC does not seem to be directly implicated in the interaction with LptBFG, as deletion of this domain impairs neither LPS biogenesis nor LptC binding to the IM complex [28]. Therefore, the LptFG periplasmic domains might be implicated in forming the periplasmic bridge of the Lpt machine. Moreover, the role of LptC is still elusive. Its C-terminal end, which is thought to interact with the N-terminus of LptA, appears to be dispensable, at least under conditions of non-limiting LptB expression; on the contrary, point mutations in the same region are lethal and are not suppressed by LptB overexpression [22,28,41]. It is also remarkable that *E*. *coli*-*P*. *aeruginosa* hybrid Lpt machines are functional. The *lptA* homologue from *P*. *aeruginosa*, *lptH*, complements *E*. *coli* Δ*lptA* mutants [29]; likewise, *P*. *aeruginosa lptC* complements *E*. *coli* Δ*lptC*, albeit an increased *lptB* expression level is required [41]. Although both homologous couples of *E*. *coli* and *P*. *aeruginosa* proteins exhibit the β-jellyroll structure, their amino acid sequence identity is scanty, thus suggesting that interactions between structural features, rather than specific amino acids, play a predominant role in the interactions between the periplasmic protein domains of the complex

Given that i) *Ec*-LptA and *Ec*-LptC can be functionally replaced by the structurally similar but scarcely conserved (as far as the amino acid sequence is concerned) *Pa*-LptH and *Pa*-LptC, respectively, and ii) the LptC N-terminal transmembrane domain is dispensable (and thus the periplasmic β-jellyroll of LptC appears to be sufficient to carry out LptC function), we addressed whether LptA and LptC could replace each other by testing complementation of *E*. *coli* bearing a chromosomal deletion of both genes (Δ*lptCA*) with plasmids expressing *lptC* or *lptA* alone. Overall the genetic data we have obtained indicate that *E*. *coli* Δ*lptC* mutant is viable only in the presence of specific suppressor mutations in *lptF*, whereas we did not obtain viable Δ*lptA* mutants.

In keeping with the low frequency of such Δ*lptC* mutants, eleven out of eleven independent mutants thus obtained were associated with an additional mutation of LptF arginine 212, being cysteine, serine, or glycine the substituting residues. Such a complete association between the lack of LptC and LptF^R212^ mutations strongly suggests that a specific suppressor is required for viability of the Δ*lptC* mutant and that LptF is the preferred (if not the only) suppressor gene.

Genomic sequencing was performed on three non-independent mutants obtained in a first round of screening. Two of them bore different amino acid substitution (R212C and R212S, thus resulting *a posteriori* independent mutants) without any other point mutation in ORFs, relative to the parental strain; the third one bore the same *lptF*^R212S^ allele and two additional single nucleotide substitution (one of which leading to a synonymous codon) within the λp*lac*Mu insertion harbored by the parental strain. Mutations in non-coding regions were clustered within spacers of rRNA and/or tRNA operons and within pseudogenes. It is thus very unlikely that these additional variations relative to the parental strain may significantly contribute to suppress the Δ*lptC* mutation.

Although the sequencing coverage was > 99%, these observations do not completely rule out that additional mutations in regions not covered by sequencing may contribute to suppress the lethal phenotype of Δ*lptC*. However, the *lptF*^R212G^ and *lptF*^R212S^ alleles were capable to suppress lethality of conditional expression *lptC* mutants in nonpermissive conditions, thus demonstrating that i) a suppressor is necessary to overcome lethality caused by LptC depletion and ii) *lptF*^R212G^ and *lptF*^R212S^ alleles are sufficient for such suppression without any hypothetical additional mutation associated with the plasmid-shuffled isolates. It is possible, however, that the higher copy number (and likely the higher expression level) of *lptA*, harbored by the chasing plasmid, may represent an additional condition required to compensate the lack of LptC.

It remains to be elucidated whether the inability of *lptF*^R212C^ allele to suppress the lethal phenotype of conditional expression *lptC* mutants depends on the recessive nature of this mutation or by the need of an as yet discovered co-suppressor. The latter hypothesis, however, seems less likely, as suppressor mutants with the *lptF*^R212C^ allele were obtained at comparable frequency (3/11) as the other two suppressors. On the other hand, *lptF*^R212C^ appears to be the weakest suppressor as this mutant exhibits several phenotypes diagnostic of cellular defects (cold sensitivity; Fig 3 and S6 Table) or, more specifically, of altered OM functionality (LPS modification, increased permeability to toxic compounds), in particular when only *lptA* is ectopically expressed. Cold sensitivity and LPS modifications of *lptF*^R212C^ are (the former only partially) alleviated by ectopic expression of both *lptA* and *lptB*. It thus appears that at least in part the OM defects revealed by the phenotype of this suppressor mutant may depend on unbalanced expression of *lptAB*. It was shown that enhanced expression of LptB appears to stabilize *E*. *coli* Lpt complexes containing non-canonical components such as a C-terminally deleted *E*. *coli* LptC mutant or *P*. *aeruginosa* LptC [41]. It may be suggested that LptB overexpression may play a similar role in the presence of the weak *lptF*^R212C^ suppressor. Finally, *lptC* appears to restore the wild type level of permeability to toxic compounds of any *lptF*^SupC^ mutant, suggesting that the mutant LptF^SupC^ may be functional also in a seven-component Lpt machine.

The LptF R212 residue is located in a predicted large periplasmic domain (residues 122–269) connecting the 3rd and 4th transmembrane helices and a similar organization is predicted for LptG [16], but their structure has not yet been solved. The suppressor phenotype exhibited by three specific mutations of LptF R212 residue, however, highlights the relevance of the LptF periplasmic loop in the LPS transport. According to the current Lpt working model [3], in the wild type seven-component Lpt machine of *E*. *coli* the β-jellyroll domains of three different Lpt components, namely the C-terminal periplasmic domain of LptC, LptA, and the periplasmic N-terminal domain of LptD form a hydrophobic groove that accompanies LPS in its way from IM to OM. We speculate that the periplasmic loop of LptF (and LptG), in the wild type form, specifically interacts with the hydrophobic groove *via* LptC and that in the absence of LptC the connection with the hydrophobic groove is compromised. The suppressor mutations in Arg212 of LptF could restore a functional interaction with the hydrophobic groove, for example by directly binding LptA without the mediation of LptC or by recruiting an additional LptA molecule to replace LptC, thus allowing a six-component Lpt machine to be functional.

We also have shown that the presence of wild type LptC is compatible with the LptF^SupC^ proteins. It remains to be assessed, however, whether LptC can be recruited by the Lpt machine when a LptF^SupC^ protein is present (as suggested by the restoration of the wild phenotype when *lptC* is present in an *lptF*^SupC^ strain) or whether the suppressors can only assemble a six-component Lpt machine. Clarifying this point will shed light on the reciprocal interactions of the Lpt components and on the structure and mechanism of the LPS transporter.

Using the powerful plasmid shuffling technique that led to the selection of Δ*lptC* suppressors LptF^SupC^ we did not obtain Δ*lptA* clones, as all the clones selected upon shuffling with the chasing plasmid harboring *lptC* still bore a displaced *lptA* allele. It is possible that LptA plays a more fundamental role in the Lpt machine. For example, the interaction of LptA with the OM complex might require specific function(s) that cannot be fulfilled by LptC even with a suppressor mutation. Alternatively, more than one suppressor mutation would be required, thus decreasing the chance of finding a mutant.

The functionality of a six-component Lpt machine suggests a modular evolution of the LPS transport system in which a hydrophobic groove that ferries LPS through the periplasm evolved by subsequent recruitment of β-jellyroll modules connecting the IM and OM complexes.

## Supporting Information

S1 Table Bacterial strains. (PDF)

S2 Table Plasmids. (PDF)

S3 Table Oligonucleotides. (PDF)

S4 Table Reads and variants analyses of the sequenced strains as compared with *E*. *coli* BW2952. (PDF)

S5 Table Mutations in intergenic regions of parental and Δ*lptC* viable mutants. (PDF)

S6 Table Phenotypic analysis of Δ*lptC* mutants. (PDF)

S1 References (PDF)
