# Supplementary material for: The Lack of the Essential LptC Protein in the Trans-Envelope Lipopolysaccharide Transport Machine Is Circumvented by Suppressor Mutations in LptF, an Inner Membrane Component of the Escherichia coli Transporter
Source: PLoS One. 2016 Aug 16;11(8):e0161354. doi: 10.1371/journal.pone.0161354 (PMC4986956; doi:10.1371/journal.pone.0161354)
Supplement: S1 Table — (PDF) [file pone.0161354.s001.pdf]

Table S1. Bacterial strains

Strain	Parental strain	Relevant characters		Features/construction	Origin
		Chromosomal	Plasmid		
AM604	MC4100	*rpsL150*			[1]
AM604/pGS401	AM604	*rpsL150*	*bla*	by transformation	[2]
AM604/pKD46	AM604	*rpsL150*	*bla*	by transformation	[2]
AM604/pKD46/pGS104	AM604/pKD46	*rpsL150*	*ptac-lptCAB cat; bla*	by transformation	[2]
FL905	AM604	Φ(*kan araC araBp-lptC*)*1*			[3]
FL905/pGS442	FL905	Φ(*kan araC araBp-lptC*)*1*	*ptac-lptFG cat*	by transformation	This work
FL905/pGS443	FL905	Φ(*kan araC araBp-lptC*)*1*	*ptac-lptF*^{R212C}*G cat*	by transformation	This work
FL905/pGS444	FL905	Φ(*kan araC araBp-lptC*)*1*	*ptac-lptF*^{R212S}*G cat*	by transformation	This work
FL905/pGS445	FL905	Φ(*kan araC araBp-lptC*)*1*	*ptac-lptFG_lptAB cat*	by transformation	This work
FL905/pGS446	FL905	Φ(*kan araC araBp-lptC*)*1*	*ptac-lptF*^{R212C}*G_lptAB cat*	by transformation	This work
FL905/pGS447	FL905	Φ(*kan araC araBp-lptC*)*1*	*ptac-lptF*^{R212S}*G_lptAB cat*	by transformation	This work
FL905/pGS450	FL905	Φ(*kan araC araBp-lptC*)*1*	*ptac-lptF*^{R212G}*G cat*	by transformation	This work
FL905/pGS451	FL905	Φ(*kan araC araBp-lptC*)*1*	*ptac-lptF*^{R212G}*G_lptAB cat*	by transformation	This work
KG-280/pGS104	AM604/pKD46/pGS104	*rpsL150* Δ*lptCA*::*kan*	*ptac-lptCAB cat*	by gene specific mutagenesis	[2]

KG-286.01/pGS104	KG-280/pGS104/pCP20	*rpsL150* Δ*lptCA*	*ptac-lptCAB cat*	by FLP-mediated kan cassette excision	[2][a]
KG-286.02/pGS308	KG-286.01/pGS104	*rpsL150* Δ*lptCA*	*ptac-lptCA kan*	by plasmid shuffling; selection for Kan^R, screening for Cam^S	This work
KG-286.03/pGS315	KG-286.02/pGS308	*rpsL150* Δ*lptCA*	*ptac-lptCAB-tetAt cat rpsL*$^+$	by plasmid shuffling; selection for Cam^R Str^S, screening for Kan^S	This work
KG-286.04/pGS308	KG-286.03/pGS315	*rpsL150* Δ*lptCA*	*ptac-lptCA kan*	by plasmid shuffling; selection for Kan^R Str^R, screening for Cam^S	[2][a]
KG-286.05/pMBM07	KG-286.04/pGS308	*rpsL150* Δ*lptCA*	*araBp-lptCA amp rpsL*$^+$ *repA101*^{ts}	by plasmid shuffling; selection for Amp^R, screening for Str^S, 28 °C. Parental, sequenced	[2][a]
KG-286.06/pGS404	KG-286.05/pMBM07	*rpsL150* Δ*lptCA*	*ptac-lptC_lptA cat*	by plasmid shuffling; selection for Cam^R Str^R at 42 °C, screening for Amp^S	[2][a]
KG-286.10/pGS321	KG-286.05/pMBM07	*rpsL150* Δ*lptCA*	*ptac-lptA cat*	by plasmid shuffling; selection for Cam^R Str^R at 42 °C, screening for Amp^S *lptA*$^+$ Δ*lptC*	This work
KG-286.07/pGS406	KG-286.05/pMBM07	*rpsL150* *ΔlptCA*	*ptac-lptCH cat*	by plasmid shuffling; selection for CamR StrR at 42 °C, screening for AmpS lptH ΔlptA	[2][a]
KG-286.13/pGS416	KG-286.05/pMBM07	*rpsL150* Δ*lptCA*	*ptac-lptAB cat*	by plasmid shuffling; selection for Cam^R Str^R at 42 °C, screening for Amp^S *lptA*$^+$ Δ*lptC*	This work
KG-286.14/pGS416	KG-286.05/pMBM07	*rpsL150* Δ*lptCA*	*ptac-lptAB cat*	by plasmid shuffling; selection for Cam^R Str^R at 42 °C, screening for Amp^S *lptA*$^+$ Δ*lptC*	This work
KG-286.21/pGS308	KG-286.06/pGS404	*rpsL150* Δ*lptCA*	*ptac-lptCA kan*	by plasmid shuffling; selection for Kan^R, screening for Cam^S *lptC*$^+$	This work
KG-286.22/pGS305	KG-286.01/pGS104	*rpsL150* Δ*lptCA*	*ptac-lptCAB kan*	by plasmid shuffling; selection for Kan^R, screening for Cam^S *lptC*$^+$	This work

KG-292.01/pGS321	KG-286.05/pMBM07	*rpsL150* Δ*lptCA; lptF*^{R212C}	*ptac-lptA cat*	by plasmid shuffling; selection for Cam^R Str^R at 42 °C, screening for Amp^S *lptA*$^+$ Δ*lptC*. Sequenced.	This work
KG-292.02/pGS308	KG-292.01/pGS321	*rpsL150* Δ*lptCA; lptF*^{R212C}	*ptac-lptCA kan*	by plasmid shuffling; selection for Kan^R, screening for Cam^S *lptC*$^+$	This work
KG-292.03/pGS323	KG-292.01/pGS321	*rpsL150* Δ*lptCA; lptF*^{R212C}	*ptac-lptA kan*	by plasmid shuffling; selection for Kan^R, screening for Cam^S *lptC*$^-$	This work
KG-293.01/pGS321	KG-286.05/pMBM07	*rpsL150* Δ*lptCA; lptF*^{R212S}	*ptac-lptA cat*	by plasmid shuffling; selection for Cam^R Str^R at 42 °C, screening for Amp^S *lptA*$^+$ Δ*lptC*. Sequenced.	This work
KG-293.02/pGS308	KG-293.01/pGS321	*rpsL150* Δ*lptCA; lptF*^{R212S}	*ptac-lptCA kan*	by plasmid shuffling; selection for Kan^R, screening for Cam^S *lptC*$^+$	This work
KG-293.03/pGS323	KG-293.01/pGS321	*rpsL150* Δ*lptCA; lptF*^{R212C}	*ptac-lptA kan*	by plasmid shuffling; selection for Kan^R, screening for Cam^S *lptC*$^-$	This work
KG-294.01/pGS416	KG-286.05/pMBM07	*rpsL150* Δ*lptCA; lptF*^{R212S}	*ptac-lptAB cat*	by plasmid shuffling; selection for Cam^R Str^R at 42 °C, screening for Amp^S *lptA*$^+$ Δ*lptC*. Sequenced.	This work
KG-294.02/pGS305	KG-294.01/pGS416	*rpsL150* Δ*lptCA; lptF*^{R212S}	*ptac-lptCAB kan*	by plasmid shuffling; selection for Kan^R, screening for Cam^S *lptC*$^+$	This work
KG-294.03/pGS324	KG-294.01/pGS416	*rpsL150* Δ*lptCA; lptF*^{R212S}	*ptac-lptAB kan*	by plasmid shuffling; selection for Kan^R, screening for Cam^S *lptC*$^-$	This work
KG-295.01/pGS321	KG-286.05/pMBM07	*rpsL150* Δ*lptCA; lptF*^{R212G}	*ptac-lptA cat*	by plasmid shuffling; selection for Cam^R Str^R at 42 °C, screening for Amp^S *lptA*$^+$ Δ*lptC*	This work
KG-295.02/pGS308	KG-295.01/pGS321	*rpsL150* Δ*lptCA; lptF*^{R212G}	*ptac-lptCA kan*	by plasmid shuffling; selection for Kan^R, screening for Cam^S *lptC*$^+$	This work
KG-295.03/pGS323	KG-295.01/pGS321	*rpsL150* Δ*lptCA; lptF*^{R212G}	*ptac-lptA kan*	by plasmid shuffling; selection for Kan^R, screening for Cam^S *lptC*$^-$	This work

KG-296.01/pGS416	KG-286.05/pMBM07	*rpsL150* Δ*lptCA; lptF*^{R212G}	*ptac-lptAB cat*	by plasmid shuffling; selection for Cam^{R} Str^{R} at 42 °C, screening for Amp^{S} *lptA*$^{+}$ Δ*lptC*	This work
KG-296.02/pGS305	KG-296.01/pGS416	*rpsL150* Δ*lptCA; lptF*^{R212G}	*ptac-lptCAB kan*	by plasmid shuffling; selection for Kan^{R}, screening for Cam^{S} *lptC*$^{+}$	This work
KG-296.03/pGS324	KG-296.01/pGS416	*rpsL150* Δ*lptCA; lptF*^{R212G}	*ptac-lptAB kan*	by plasmid shuffling; selection for Kan^{R}, screening for Cam^{S} *lptC*$^{-}$	This work
KG-297.01/pGS416	KG-286.05/pMBM07	*rpsL150* Δ*lptCA; lptF*^{R212C}	*ptac-lptAB cat*	by plasmid shuffling; selection for Cam^{R} Str^{R} at 42 °C, screening for Amp^{S} *lptA*$^{+}$ Δ*lptC*	This work
KG-297.02/pGS305	KG-297.01/pGS416	*rpsL150* Δ*lptCA; lptF*^{R212C}	*ptac-lptCAB kan*	by plasmid shuffling; selection for Kan^{R}, screening for Cam^{S} *lptC*$^{+}$	This work
KG-297.03/pGS324	KG-297.01/pGS416	*rpsL150* Δ*lptCA; lptF*^{R212C}	*ptac-lptAB kan*	by plasmid shuffling; selection for Kan^{R}, screening for Cam^{S} *lptC*$^{-}$	This work
KG-299.01/pGS321	KG-286.05/pMBM07	*rpsL150* Δ*lptCA; lptF*^{R212G}	*ptac-lptA cat*	by plasmid shuffling; selection for Cam^{R} Str^{R} at 42 °C, screening for Amp^{S} *lptA*$^{+}$ Δ*lptC*	This work
KG-300.01/pGS321	KG-286.05/pMBM07	*rpsL150* Δ*lptCA; lptF*^{R212G}	*ptac-lptA cat*	by plasmid shuffling; selection for Cam^{R} Str^{R} at 42 °C, screening for Amp^{S} *lptA*$^{+}$ Δ*lptC*	This work
KG-301.01/pGS321	KG-286.05/pMBM07	*rpsL150* Δ*lptCA; lptF*^{R212S}	*ptac-lptA cat*	by plasmid shuffling; selection for Cam^{R} Str^{R} at 42 °C, screening for Amp^{S} *lptA*$^{+}$ Δ*lptC*	This work
KG-302.01/pGS416	KG-286.05/pMBM07	*rpsL150* Δ*lptCA; lptF*^{R212S}	*ptac-lptAB cat*	by plasmid shuffling; selection for Cam^{R} Str^{R} at 42 °C, screening for Amp^{S} *lptA*$^{+}$ Δ*lptC*	This work
KG-303.01/pGS416	KG-286.05/pMBM07	*rpsL150* Δ*lptCA; lptF*^{R212C}	*ptac-lptAB cat*	by plasmid shuffling; selection for Cam^{R} Str^{R} at 42 °C, screening for Amp^{S} *lptA*$^{+}$ Δ*lptC*	This work

NR1113	NR754	Δ(λatt-lom)::*bla* *araBp-lptFG* Δ*lptFG*			[4]
NR1113/pGS401	NR1113	*araBp-lptFG*	*ptac*-void	by transformation	This work
NR1113/pGS442	NR1113	*araBp-lptFG*	*ptac-lptFG cat*	by transformation	This work
NR1113/pGS443	NR1113	*araBp-lptFG*	*ptac-lptF*^{R212C}*G cat*	by transformation	This work
NR1113/pGS444	NR1113	*araBp-lptFG*	*ptac-lptF*^{R212S}*G cat*	by transformation	This work
NR1113/pGS450	NR1113	*araBp-lptFG*	*ptac-lptF*^{R212G}*G cat*	by transformation	This work

[a] KG-286/pGS104 parental strain and its derivatives described by [2] have been renumbered by adding decimal figures to the host collection name just to distinguish individual KG-286 derivatives, as they may have acquired different mutations upon selection by plasmid shuffling.
