# Supplementary material for: The Lack of the Essential LptC Protein in the Trans-Envelope Lipopolysaccharide Transport Machine Is Circumvented by Suppressor Mutations in LptF, an Inner Membrane Component of the Escherichia coli Transporter
Source: PLoS One. 2016 Aug 16;11(8):e0161354. doi: 10.1371/journal.pone.0161354 (PMC4986956; doi:10.1371/journal.pone.0161354)
Supplement: S2 Table — (PDF) [file pone.0161354.s002.pdf]

Table S2. Plasmids

Plasmids	Parental / replicon	Relevant characters	Construction/Origin
pCP20		*bla, cat,* thermosensitive replication	[5]
pGS100	pGZ119EH	*ptac*-TIR, *cat, oriV*$_{\mathrm{ColD}}$	[6]
pGS104	pGS100	*ptac-lptCAB, cat, oriV*$_{\mathrm{ColD}}$	[6]
pGS105	pGS100	*ptac-lptAB, cat, oriV*$_{\mathrm{ColD}}$	[6]
pGS303	pGS100	*kan*	[2]
pGS305	pGS303	*ptac-lptCAB, kan, oriV*$_{\mathrm{ColD}}$	*lptCAB* was obtained by *Eco*RI-*Xba*I digestion of pGS104 and cloned into pGS303 *Eco*RI-*Xba*I sites
pGS306	pGS100	*ptac-lptCA, cat, oriV*$_{\mathrm{ColD}}$	*lptCA* was PCR-amplified with AP54-FG2723 primers from pGS104 and cloned into *EcoR*I-*Xba*I sites of pGS100
pGS308	pGS303	*ptac-lptCA, kan, oriV*$_{\mathrm{ColD}}$	[2]
pGS321	pGS100	*ptac-lptA, cat, oriV*$_{\mathrm{ColD}}$	*lptA* was PCR-amplified with AP55-FG2723 primers from pGS104 and cloned into *EcoR*I-*Xba*I sites of pGS100
pGS323	pGS303	*ptac-lptA, kan, oriV*$_{\mathrm{ColD}}$	*lptA* was obtained by *Eco*RI-*Xba*I digestion of pGS321 and cloned into pGS303 *Eco*RI-*Xba*I sites
pGS324	pGS303	*ptac-lptAB, kan, oriV*$_{\mathrm{ColD}}$	*lptAB* was obtained by *Eco*RI-*Xba*I digestion of pGS105 and cloned into pGS303 *Eco*RI-*Xba*I sites
pGS401	pGS100	*ptac*-SD1-*Eco*RI-*XbaI*-SD2-*SalI*-*Hin*dIII, *cat, oriV*$_{\mathrm{ColD}}$	[2]
pGS402	pGS401	*ptac-lptC, cat, oriV*$_{\mathrm{ColD}}$	[2]
pGS404	pGS402	*ptac-lptC-lptA, cat, oriV*$_{\mathrm{ColD}}$	[2]
pGS406	pGS401	*ptac-lptCH, cat, oriV*$_{\mathrm{ColD}}$	[2]
pGS416	pGS401	*ptac-lptAB, cat, oriV*$_{\mathrm{ColD}}$	[2]
pGS420	pGS401	*ptac-* $malE_{SS}lptC^{\Delta 1-23}$, *cat, oriV*$_{\mathrm{ColD}}$	$malE_{SS}lptC^{\Delta 1-23}$ was amplified by three step PCR with FG3089, AP211, AP212 and FG3090 primers from AM604 genomic DNA and cloned into *Eco*RI-*Xba*I sites of pGS401

pGS442	pGS401	ptac-*lptFG*, *cat, oriV*$_{ColD}$	*lptFG* genes were PCR-amplified with FG3195-FG3196 primers from KG-286.05/pMBM07 genomic DNA and cloned into *Eco*RI-*Xba*I sites of pGS401 downstream of SD1
pGS443	pGS401	*ptac-lptF*^{R212C}*G*, *cat, oriV*$_{ColD}$	*lptF*^{R212C}*G* genes were PCR-amplified with FG3195-FG3196 primers from KG-292.01/pGS321 genomic DNA and cloned into *Eco*RI-*Xba*I sites of pGS401 downstream of SD1
pGS444	pGS401	*ptac-lptF*^{R212S}*G*, *cat, oriV*$_{ColD}$	*lptF*^{R212S}*G* genes were PCR-amplified with FG3195-FG3196 primers from KG-293.01/pGS321 genomic DNA and cloned into *Eco*RI-*Xba*I sites of pGS401 downstream of SD1
pGS445	pGS416	*ptac-lptFG_lptAB*, *cat, oriV*$_{ColD}$	*lptFG* genes were PCR-amplified with FG3195-FG3196 primers from KG-286.05/pMBM07 genomic DNA and cloned into *Eco*RI-*Xba*I sites of pGS416 downstream of SD1
pGS446	pGS416	*ptac-lptF*^{R212C}*G_lptAB*, *cat, oriV*$_{ColD}$	*lptF*^{R212C}*G* genes were PCR-amplified with FG3195-FG3196 primers from KG-292.01/pGS321 genomic DNA and cloned into *Eco*RI-*Xba*I sites of pGS416 downstream of SD1
pGS447	pGS416	*ptac-lptF*^{R212S}*G_lptAB*, *cat, oriV*$_{ColD}$	*lptF*^{R212S}*G* genes were PCR-amplified with FG3195-FG3196 primers from KG-293/pGS321 genomic DNA and cloned into *Eco*RI-*Xba*I sites of pGS416 downstream of SD1
pGS450	pGS401	ptac-*lptF*^{R212G}*G*, *cat, oriV*$_{ColD}$	*lptF*^{R212G}*G* genes were PCR-amplified with FG3195-FG3196 primers from KG-293/pGS321 genomic DNA and cloned into *Eco*RI-*Xba*I sites of pGS401 downstream of SD1
pGS451	pGS416	*ptac-lptF*^{R212G}*G_lptAB*, *cat, oriV*$_{ColD}$	*lptF*^{R212G}*G* genes were PCR-amplified with FG3195-FG3196 primers from KG-295.01/pGS321 genomic DNA and cloned into *Eco*RI-*Xba*I sites of pGS416 downstream of SD1
pKD46		*oriR101, repA101ts, araC, araBp-λ red, bla*	[5]
pMBM07	pKD46	*araBp-lptCA, rpsL*$^{+}$*, bla, oriR101, repA101ts*	[2]
