# Supplementary material for: The Lack of the Essential LptC Protein in the Trans-Envelope Lipopolysaccharide Transport Machine Is Circumvented by Suppressor Mutations in LptF, an Inner Membrane Component of the Escherichia coli Transporter
Source: PLoS One. 2016 Aug 16;11(8):e0161354. doi: 10.1371/journal.pone.0161354 (PMC4986956; doi:10.1371/journal.pone.0161354)
Supplement: S3 Table — (PDF) [file pone.0161354.s003.pdf]

Table S3. Oligonucleotides

Name	Sequence[a]	Notes
AP54	cgagaggaattcaccATGAGTAAAGCCAGACGTTGGG	pGS306 construction with FG2723; *Eco*RI
AP55	cgagagaggaattcaacATGAAATTCAAAACAAACAAACTC	pGS321 construction with FG2723; *Eco*RI
AP211	GTATCGTCTTTTTCGGCCATGGCGAGAGCCGAGGCGGAAAAC	pGS420 construction with AP212, FG3089 and FG3090
AP212	GTTTTCCGCCTCGGCTCTCGCCATGGCCGAAAAAGACGATAC	pGS420 construction with AP211, FG3089 and FG3090
FG2723	gactagtctagaTTAATTACCCTTCTTCTGTGCCGGGG	pGS306 and pGS321 construction with AP54 and AP55; *Xba*I
FG3089	catattcgtctcgaattcaccATGAAAATAAAAACAGGTGCACGC	pGS420 construction with AP211, AP212 and FG3090; *Esp*3I-*Eco*RI
FG3090	caggttcgtctctctagaTTAAGGCTGAGTTTGTTTGTTTTG	pGS420 construction with AP211, AP212 and FG3089; *Esp*3I-*Xba*I
FG3129	AGTAAAGCCAGACGTTGGG	Southern blotting *lptC* probe amplification by PCR
FG3130	CCTTTTCAATCAGCTCGGC	Southern blotting *lptC* probe amplification by PCR
FG3195	gataggaattcaccGTGATAATCATAAGATATCTGG	pGS442, pGS443, pGS444, pGS445, pGS446, pGS447, pGS450 and pGS451 construction with FG3196; *Eco*RI
FG3196	ggctagtctagaTTACGATTTTCTCATTAACAGC	pGS442, pGS443, pGS444, pGS445, pGS446, pGS447, pGS450 and pGS451 construction with FG3195; *Xba*RI

[a] Upper case letters, sequence present in the template; lower case letters, additional/modified sequence not present in the template; restriction sites are underlined.
