# Supplementary material for: The Lack of the Essential LptC Protein in the Trans-Envelope Lipopolysaccharide Transport Machine Is Circumvented by Suppressor Mutations in LptF, an Inner Membrane Component of the Escherichia coli Transporter
Source: PLoS One. 2016 Aug 16;11(8):e0161354. doi: 10.1371/journal.pone.0161354 (PMC4986956; doi:10.1371/journal.pone.0161354)
Supplement: S4 Table — (PDF) [file pone.0161354.s004.pdf]

Table S4. Reads and variants analyses of the sequenced strains as compared with *E. coli* BW2952

Sequenced strain[a]:	A	B	C	D
**Sequencing mapping**				
Number of raw reads	6,901,208	6,797,355	6,749,884	6,461,256
Mapped reads	6,580,797	6,462,280	6,391,090	6,162,089
Unmapped reads	320,411	335,075	358,794	299,167
Mapping rate (%)	95.36%	95.07%	94.68%	95.37%
Min/max/mean read length	35/301/280.69	35/301/278.59	35/301/282.47	35/301/283.4
Mean sequencing depth[b] (fold)	391.71	381.73	383.11	370.70
Coverage[c] (%)	99.10	99.20	98.82	99.10
**Variants analysis**				
Total High Quality Variants	34	35	36	33
Variants rate	134.651	130.804	127.171	138.732
Raw INDEL variants	7	8	7	7
Raw SNV variants	27	27	29	26
Total variants in intergenic_regions	23	21	23	21
Total frameshift_variants	2	2	2	2
Total missense_variants	2	3	4	3
Total synonymous_variants	0	1	0	0

[a] A, KG-286.05/pMBM07 (parental); B, KG-292.01/pGS321; C, KG-293.01/pGS321; D, KG-294.01/pGS416

[b] Mean depth=MR*MRL/GL, where MR= Number of mapped reads, MRL= Mean reads length, GL= Reference genome length

[c] Coverage (%) = CN*100/GL, where CN = Number of nucleotides covered by at least one read, GL= Reference genome length
