# Supplementary material for: The Lack of the Essential LptC Protein in the Trans-Envelope Lipopolysaccharide Transport Machine Is Circumvented by Suppressor Mutations in LptF, an Inner Membrane Component of the Escherichia coli Transporter
Source: PLoS One. 2016 Aug 16;11(8):e0161354. doi: 10.1371/journal.pone.0161354 (PMC4986956; doi:10.1371/journal.pone.0161354)
Supplement: S5 Table — (PDF) [file pone.0161354.s005.pdf]

Table S5. Mutations in intergenic regions of parental and Δ*lptC* viable mutants[a]

STRAIN[b]	Position[c]		Nucleotide change	Left gene[de]	Right gene[de]	Notes[e]
**B**	683,472		A	*lysZ*	*lysQ**	
**B**	683,474		G	*lysZ*	*lysQ**	
**B**	683,636		G	*lysQ**	*lysQ*	
**BCD**	683,681		C	*lysQ**	*lysQ*	
**BCD**	683,682		C	*lysQ**	*lysQ*	
**BCD**	683,695		T	*lysQ**	*lysQ*	
**BCD**	683,735		A	*lysQ**	*lysQ*	
**BCD**	683,736		T	*lysQ**	*lysQ*	
**BCD**	683,739		T	*lysQ**	*lysQ*	
**BCD**	683,742		T	*lysQ**	*lysQ*	
**A**	683,764		-	*lysQ**	*lysQ*	
**B**	683,765	- 683,766	GGTAACACCCGT	*lysQ**	*lysQ*	
**D**	683,767		T	*lysQ**	*lysQ*	
**C**	1,288,805		-	*fnr*	*ogt*	within *insH**
**D**	1,288,862	- 1,288,864	C	*fnr*	*ogt*	within *insH**
**BCD**	1,289,316	- 1,289,318	A	*fnr*	*ogt*	within *insH**
**A**	4,056,058	- 4,056,066	TTT	*rrsB*	*rrlB*	within *gltT**
**A**	4,056,061		-	*rrsB*	*rrlB*	within *gltT**
**D**	4,056,210		A	*gltT**	*rrlB*	

[a] 53 mutations shared by parental and mutants relative to the reference BW2952 sequence are not reported.

[b] A, KG-286.05/pMBM07; B, KG-292.01/pGS321; C, KG-293.01/pGS321; D, KG-294.01/pGS416.

[c] Mutation occurred at the given coordinate or within the range indicated.

[d] Genes delimiting at the left and right the intergenic region are reported.

[e] Asterisk (*) denotes pseudogenes
