# Supplementary material for: The Lack of the Essential LptC Protein in the Trans-Envelope Lipopolysaccharide Transport Machine Is Circumvented by Suppressor Mutations in LptF, an Inner Membrane Component of the Escherichia coli Transporter
Source: PLoS One. 2016 Aug 16;11(8):e0161354. doi: 10.1371/journal.pone.0161354 (PMC4986956; doi:10.1371/journal.pone.0161354)
Supplement: S6 Table — (PDF) [file pone.0161354.s006.pdf]

Table S6. Phenotypic analysis of Δ*lptC* mutants[a]

Strain	*lptF* allele	*lpt* alleles on plasmid	15°C	42°C	Bac	Nov	Rif	SDS EDTA	McC
KG-286.21/pGS308	wt	p*lptCA*	+	+	+	+	+	+	+
KG-286.22/pGS305	wt	p*lptCAB*	+	+	+	+	+	+	+
KG-292.03/pGS323	R212C	p*lptA*	-	+	-	-	-	-	-
KG-297.03/pGS324	R212C	p*lptAB*	±	+	-	-	-	-	-
KG-292.02/pGS308	R212C	p*lptCA*	+	+	+	+	+	+	+
KG-297.02/pGS305	R212C	p*lptCAB*	+	+	+	+	+	+	+
KG-295.03/pGS323	R212G	p*lptA*	+	+	+	-	+	±	±
KG-296.03/pGS324	R212G	p*lptAB*	+	+	+	±	+	+	+
KG-295.02/pGS308	R212G	p*lptCA*	+	+	+	+	+	+	+
KG-296.02/pGS305	R212G	p*lptCAB*	+	+	+	+	+	+	+
KG-293.03/pGS323	R212S	p*lptA*	+	+	+	-	+	±	-
KG-294.03/pGS324	R212S	p*lptAB*	+	+	+	±	+	+	+
KG-293.02/pGS308	R212S	p*lptCA*	+	+	+	+	+	+	+
KG-294.02/pGS305	R212S	p*lptCAB*	+	+	+	+	+	+	+

[a] Efficiency of plating relative to growth in LB-glucose-kanamicin at 37 °C is indicated. +, between 1 and 0.1; ±, between 0.1 and $3x10^{-2}$; -, < $3x10^{-2}$. Bct, 50 µg/ml bacitracin; Nov, 10 µg/ml novobiocin; Rif, 2.5µg/ml rifampicin, SDS-EDTA, 0.5% and 0.25 mM, respectively; McC, MacConkey-glucose-chloramphenicol. The experiment was performed as described in legend of Fig 3.
