# Supplementary material for: The Lack of the Essential LptC Protein in the Trans-Envelope Lipopolysaccharide Transport Machine Is Circumvented by Suppressor Mutations in LptF, an Inner Membrane Component of the Escherichia coli Transporter
Source: PLoS One. 2016 Aug 16;11(8):e0161354. doi: 10.1371/journal.pone.0161354 (PMC4986956; doi:10.1371/journal.pone.0161354)
Supplement: S1 References — (PDF) [file pone.0161354.s007.pdf]

S1 References

1. Wu T, McCandlish AC, Gronenberg LS, Chng SS, Silhavy TJ, et al. (2006) Identification of a protein complex that assembles lipopolysaccharide in the outer membrane of *Escherichia coli*. Proc Natl Acad Sci USA 103: 11754-11759.
2. Bollati M, Villa R, Gourlay LJ, Benedet M, Dehò G, et al. (2015) Crystal structure of LptH, the periplasmic component of the lipopolysaccharide transport machinery from *Pseudomonas aeruginosa*. FEBS J 282: 1980-1997.
3. Sperandeo P, Lau FK, Carpentieri A, De Castro C, Molinaro A, et al. (2008) Functional analysis of the protein machinery required for transport of lipopolysaccharide to the outer membrane of *Escherichia coli*. J Bacteriol 190: 4460-4469.
4. Ruiz N, Gronenberg LS, Kahne D, Silhavy TJ (2008) Identification of two inner-membrane proteins required for the transport of lipopolysaccharide to the outer membrane of *Escherichia coli*. Proc Natl Acad Sci USA 105: 5537-5542.
5. Datsenko KA, Wanner BL (2000) One-step inactivation of chromosomal genes in *Escherichia coli* K-12 using PCR products. Proc Natl Acad Sci USA 97: 6640-6645.
6. Sperandeo P, Pozzi C, Dehò G, Polissi A (2006) Non-essential KDO biosynthesis and new essential cell envelope biogenesis genes in the *Escherichia coli yrbG-yhbG* locus. Res Microbiol 157: 547-558.
